# Comparison of intergenerational instrumental variable analyses of body mass index and mortality in UK Biobank

**DOI:** 10.1093/ije/dyac159

**Published:** 2022-08-10

**Authors:** Ciarrah-Jane Barry, David Carslake, Kaitlin H Wade, Eleanor Sanderson, George Davey Smith

**Affiliations:** Medical Research Council (MRC) Integrative Epidemiology Unit at the University of Bristol, Bristol, UK; Population Health Sciences, Bristol Medical School, Faculty of Health Sciences, University of Bristol, Bristol, UK; Department of Mathematical Sciences, University of Bath, Bath, UK; Medical Research Council (MRC) Integrative Epidemiology Unit at the University of Bristol, Bristol, UK; Population Health Sciences, Bristol Medical School, Faculty of Health Sciences, University of Bristol, Bristol, UK; Medical Research Council (MRC) Integrative Epidemiology Unit at the University of Bristol, Bristol, UK; Population Health Sciences, Bristol Medical School, Faculty of Health Sciences, University of Bristol, Bristol, UK; Medical Research Council (MRC) Integrative Epidemiology Unit at the University of Bristol, Bristol, UK; Population Health Sciences, Bristol Medical School, Faculty of Health Sciences, University of Bristol, Bristol, UK; Medical Research Council (MRC) Integrative Epidemiology Unit at the University of Bristol, Bristol, UK; Population Health Sciences, Bristol Medical School, Faculty of Health Sciences, University of Bristol, Bristol, UK

**Keywords:** Mendelian randomization, instrumental variable, proxy genotype, offspring as instrument, intergenerational, body mass index, mortality, UK Biobank

## Abstract

**Background:**

An increasing proportion of people have a body mass index (BMI) classified as overweight or obese and published studies disagree whether this will be beneficial or detrimental to health. We applied and evaluated two intergenerational instrumental variable methods to estimate the average causal effect of BMI on mortality in a cohort with many deaths: the parents of UK Biobank participants.

**Methods:**

In Cox regression models, parental BMI was instrumented by offspring BMI using an ‘offspring as instrument’ (OAI) estimation and by offspring BMI-related genetic variants in a ‘proxy-genotype Mendelian randomization’ (PGMR) estimation.

**Results:**

Complete-case analyses were performed in parents of 233 361 UK Biobank participants with full phenotypic, genotypic and covariate data. The PGMR method suggested that higher BMI increased mortality with hazard ratios per kg/m^2^ of 1.02 (95% CI: 1.01, 1.04) for mothers and 1.04 (95% CI: 1.02, 1.05) for fathers. The OAI method gave considerably higher estimates, which varied according to the parent–offspring pairing between 1.08 (95% CI: 1.06, 1.10; mother–son) and 1.23 (95% CI: 1.16, 1.29; father–daughter).

**Conclusion:**

Both methods supported a causal role of higher BMI increasing mortality, although caution is required regarding the immediate causal interpretation of these exact values. Evidence of instrument invalidity from measured covariates was limited for the OAI method and minimal for the PGMR method. The methods are complementary for interrogating the average putative causal effects because the biases are expected to differ between them.

Key Messages

- This study used two complementary methods to estimate the causal effect of body mass index (BMI) on mortality in the parents of a large sample of individuals from UK Biobank.
- Offspring BMI and BMI-related genotype were used as two separate intergenerational instruments to obtain causal estimates without some of the limitations of conventional observational analyses. Instrument–exposure associations were derived from independent samples (the 1958 birth cohort and the Genetic Investigation of ANthropometric Traits consortium, respectively).
- Results support a causal role of higher BMI increasing mortality but estimates using an offspring’s BMI to proxy for parent’s BMI may be biased by differences in socio-demographic characteristics between the two samples used.
- Both methods are of value, and are indeed complementary, when interrogating putative causal effects on mortality as they each have their own strengths and limitations.
- This study provides important evidence to support the use of a combination of methods to strengthen causal inference.

## Introduction

The high global prevalence of obesity, characterized as a body mass index (BMI) of >30 kg/m^2^, has increased interest in the pattern and causal nature of the relationship between BMI and mortality. Some literature shows elevated mortality at both extremes of BMI;^1^^,^^2^ however the precise shape and meaning of this association is disputed. Additionally, there is inconsistent evidence on the optimal BMI for survival, with some studies suggesting it falls within the ‘overweight’ range (25–30 kg/m^2^).^3^ These studies are mainly observational, population-based investigations and thus are subject to numerous limitations. For example, BMI measured at a single time is assumed to reflect an individual’s typical weight, but BMI may be influenced by ill-health (i.e. reverse causation).^4^ Furthermore, height and weight are prone to measurement error (particularly when self-reported or recalled), which could bias associations towards the null.^5^ There is also considerable potential for confounding in either direction, acting at different parts of the BMI distribution.

Instrumental variables (IVs) estimation can be used to obtain estimates of the average causal effects of putative exposures that are free from bias due to confounding, reverse causation or measurement error, provided certain assumptions hold. An IV should be associated with the exposure of interest, it should be independent of the outcome (except via the exposure) and there should be no confounding of the association between the IV and outcome.^6–9^ Therefore, any association between a valid IV and the outcome provides evidence of a causal relationship between the exposure and outcome and, with certain additional assumptions, a linear estimate can be made of the effect size. Two commonly used IVs are (i) exposures measured in an individual’s offspring, henceforth termed the ‘offspring as instrument’ (OAI) method,^10–12^ and (ii) genetic variants associated with the exposure, a method known as Mendelian randomization (MR).^12–14^ Existing research has suggested a linear intergenerational association of offspring BMI with parental mortality and OAI estimates of the effect of BMI that exceed conventional observational ones.^12^^,^^15^ This method is less susceptible to reverse causation, a major distorting factor within observational studies of the relationship between BMI and mortality, and has demonstrated seemingly improved causal inference in intergenerational studies.^15^^,^^16^ Within a given sample size, OAI has higher statistical power than MR.^17^ However, some confounding factors, such as socio-economic position, are likely to be intergenerationally associated, biasing OAI estimates of BMI effects.^18^

**Figure 1:**
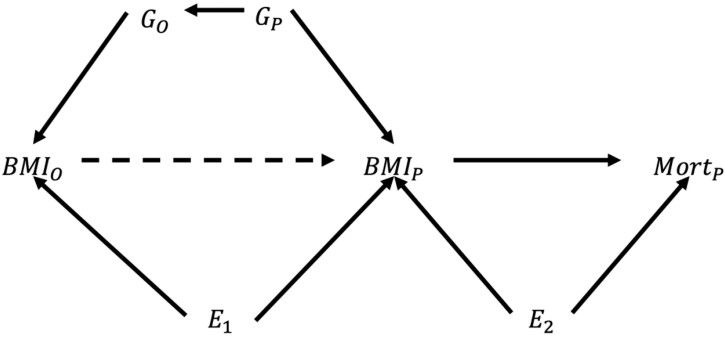
Illustration of the probable causal relationships explored in these analyses. The dashed arrow indicates an absent causal relationship. A conventional observational analysis of the effect of parental body mass index (BMI) (*BMI_P_*) on parental mortality (*Mort_P_*) would be confounded by their common environmental causes (*E_2_*). For an unbiased instrumental variables analysis, *BMI_P_* must be a collider on any pathway between the instrument and *Mort_P_* that does not include the causal effect of *BMI_P_* on *Mort_P_*. This means that the instrument must cause *BMI_P_* or they must have a common cause. If *BMI_P_* causes the instrument, estimates will be biased. It is not plausible that the instrument causes *BMI_P_* (dotted line) in either a proxy-genotype Mendelian randomization (PGMR) or an offspring as instrument (OAI) analysis. In a PGMR analysis, the instrument is the offspring’s genotype (G_*o*_). This is not plausibly caused by *BMI_P_* but is associated with it due to their common cause being parental genotype (G_*p*_). Furthermore, G_*p*_ is likely to be independent of *E_2_*, making G_*o*_ a valid instrument for *BMI_P_*. In an OAI analysis, the instrument is the offspring’s BMI (*BMI_o_*). We must assume that parental BMI does not have a causal effect on offspring BMI but that they are associated due to common genetic (G_*P*_) and environmental (*E_1_*) causes. As discussed above, the common genetic causes are plausibly independent of *E_2_* but we cannot establish whether *E_1_* and *E_2_* are independent; non-independence of *E_1_* and *E_2_* would invalidate *BMI_O_* as an instrument

In contrast, MR uses one or more genetic variants associated with the exposure as IVs, assuming the effect size of the genetic variant on the outcome is predictable. Where multiple genetic variants are available, they may be combined into a genetic risk score (GRS) and used as a single IV or used individually with estimates subsequently combined. During gamete formation, genetic variants are approximately randomly allocated^13^^,^^19^ and an individual’s genetic variants are considerably less likely to be associated with any confounding variables than are measured exposures and outcomes.^20^^,^^21^ Moreover, genetic variants estimate the effects of long-term predisposition to the exposure^19^^,^^22^ and so estimate the effect of lifelong genetic liability to the exposure,^23^ reducing the impact of measurement error and improving the accuracy and reliability of findings.^18^^,^^24^^,^^25^ Cohorts with extensive genotyping are often too recent to have had many deaths, limiting the power of conventional MR analyses to estimate the effects of putative risk factors on mortality. Where the longevity of the cohort’s parents is recorded, an extension of MR can be used to analyse survival outcomes in the parental generation using genetic variation in cohort members as a proxy genotype—henceforth termed ‘proxy-genotype Mendelian randomization’ (PGMR)—exploiting the predictable genetic association between generations (Figure 1).^26^

In this study, we compared the results obtained and potential biases from two different intergenerational methods for estimating the linear effect of BMI on mortality. The approaches we consider are: (i) the OAI method instrumenting parental BMI with offspring BMI and (ii) PGMR methods instrumenting parental BMI with the offspring’s BMI-associated genetic variants. This analysis was facilitated by the large sample of phenotypic, genetic and parental data available within UK Biobank.

## Methods

### The UK Biobank study

Cross-sectional baseline data were used from UK Biobank, a large-scale population-based study of 502 528 individuals, predominantly aged 40–69 years, between 2006 and 2010 to aid studies of human diseases in middle-aged and older individuals.^27^^,^^28^ UK Biobank was approved by the Northwest Research Ethics committee. Each participant’s weight and height were recorded, a blood sample was taken and they were invited to complete a touchscreen questionnaire with questions about health, socio-demographic, environmental and lifestyle factors.^29^ The questionnaire also asked how old (in whole years) the participant’s parents were, or how old they were when they died, providing the outcome for the survival analyses.

**Figure 2:**
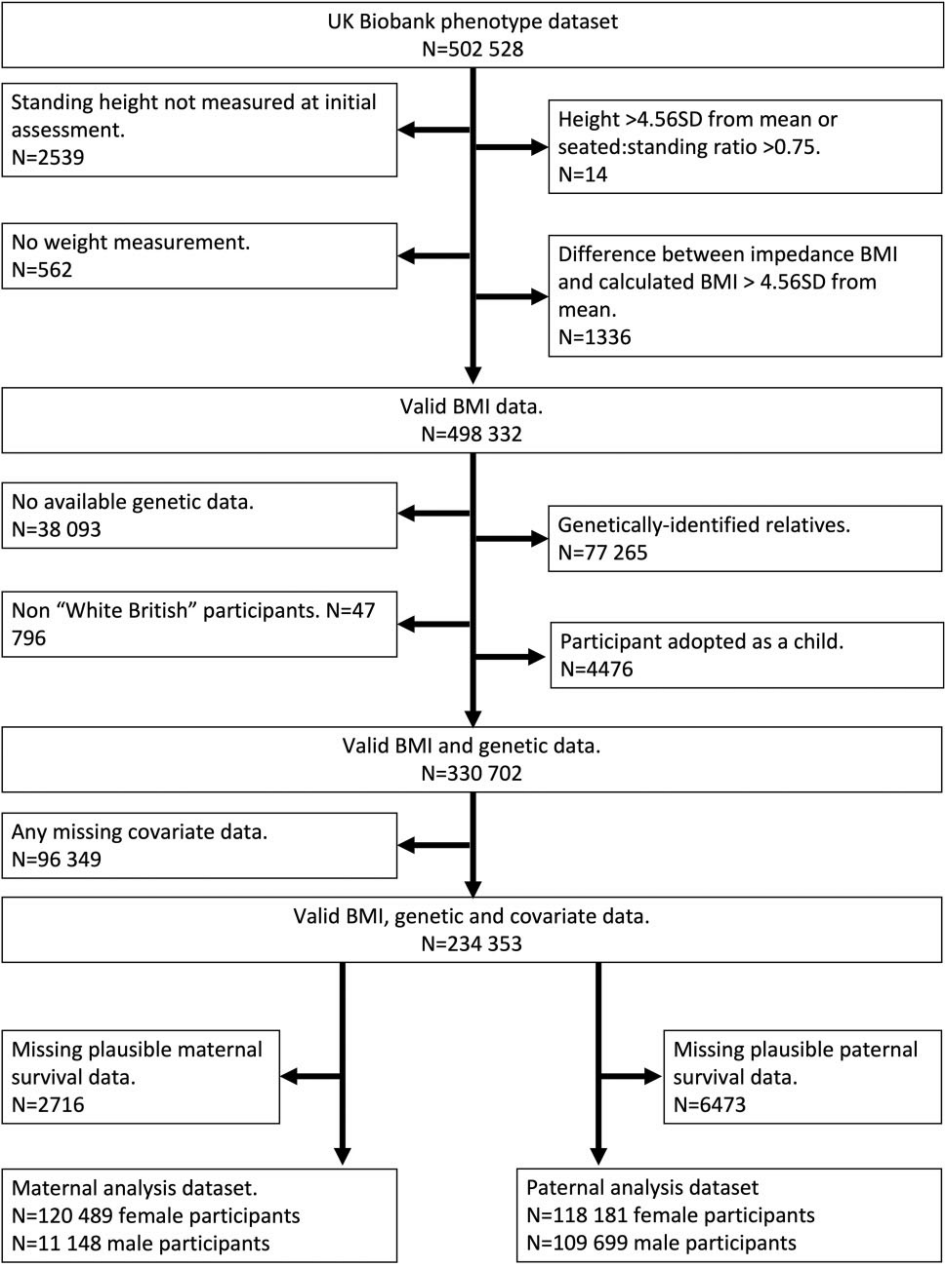
Flowchart of participants included in main analyses. BMI, body mass index

A variety of techniques measured weight, culminating into a single weight variable in the UK Biobank data set, and both standing and seated height were measured using a Seca 202 device. Two measures of BMI were available: a manual calculation of weight divided by height squared (kg/m^2^) and an electrical impedance measure of mass (kg), which was used to verify conventional BMI and to replace it when missing (Figure 2).

Baseline questionnaire items used in the present study were the participant’s age, year of birth, month of birth, sex, ethnicity, adoption status, current employment status, education, physical activity, tobacco and alcohol use and household income. Participants were also asked if their mothers smoked at the time of their birth. Full details of the classification of these are given in the Supplementary methods (available as Supplementary data at *IJE* online) and the UK Biobank variable codes are given in Supplementary Table S1 (available as Supplementary data at *IJE* online).

Genotyping of the UK Biobank blood samples has been described elsewhere.^28^^,^^30^ Genetic data were used to confirm participants’ sex and ethnicity and to identify third-degree or closer relatives. For the main analyses, data were used on 97 single-nucleotide polymorphisms (SNPs) found to be associated with BMI at *P* < 5 × 10^–8^ in the ‘most significant analysis’ of a genome-wide association study (GWAS) conducted by the Genetic Investigation of ANthropometric Traits (GIANT) consortium.^31^ For each UK Biobank participant, the average number of BMI-increasing alleles over the 97 SNPs was calculated, weighting the dosage of each genetic variant by the per-allele effect on BMI in the ‘most significant analysis’ from GIANT (Supplementary Table S2, available as Supplementary data at *IJE* online). A GRS for BMI was calculated by multiplying this weighted average by the number of variants (i.e. 97), making it a weighted total number of BMI-increasing alleles. Further details of the processing of the genetic data are given in the Supplementary methods (available as Supplementary data at *IJE* online).

Participants were excluded if outcome, instrument or covariate data were missing. The analysis was restricted to unrelated participants of self-reported White British ethnicity and genetically confirmed White European ancestry who were not adopted. Full details of the selection process are in the Supplementary methods (available as Supplementary data at *IJE* online) and Figure 2.

### Statistical analysis

Covariate associations with BMI and the GRS in UK Biobank participants were assessed using separate linear regression models. For BMI these were unadjusted, and for the GRS they were adjusted for the first 10 genetic principal components. Covariate associations with parental survival were assessed using separate Cox proportional hazards regression models adjusted only for the offspring’s (i.e. UK Biobank participant’s) date of birth.

The average causal effect of BMI on mortality in the parents of the UK Biobank participants was estimated using the IV ratio method, with offspring BMI (i.e. the OAI method) or offspring GRS (GRS-PGMR) used as instruments for parental BMI. The numerator of this method is the association between the outcome and the instrument, and the denominator is the association between the exposure and the instrument. Full details of the approach are given in the Supplementary methods (available as Supplementary data at *IJE* online).

The numerators for the causal estimates were estimated using Cox proportional hazards regression models of parental mortality against each instrument. Analyses were automatically adjusted for age using a time axis of parental age. Follow-up was left-truncated at the offspring’s birth and right-censored at their time of assessment. Analyses for both methods were first conducted ‘unadjusted’, adjusting only for the offspring’s date of birth to represent linear secular trends. All PGMR analyses (i.e. the GRS-PGMR and the summary-PGMR described below) were additionally adjusted for the first 10 genetic principal components. Adjusted models additionally adjusted for education, employment, smoking, alcohol consumption, physical activity and household income—common confounders of the BMI–mortality relationship.^32^ These were all measured in the offspring and treated as categorical variables except date of birth and physical exercise (see Supplementary methods, available as Supplementary data at *IJE* online for details of categories). Mothers and fathers were analysed separately. As the association between offspring BMI and parental BMI (but not offspring genotype and parental BMI) could plausibly differ by offspring sex, we analysed sons and daughters separately for the OAI analyses and combined the results through meta-analysis.

For the OAI method, the denominators of the causal estimates were estimated from intergenerational associations (IGAs) for BMI. Multiple estimates were obtained by a review of the literature (Supplementary Table S3, available as Supplementary data at *IJE* online). We employed estimates from the 1958 British birth cohort,^33^ which was the closest demographic match to UK Biobank participants. This supplied IGA estimates for each sex-specific combination between offspring and parents, alongside the SDs of BMI necessary to invert the associations (see Supplementary methods, available as Supplementary data at *IJE* online). For the GRS-PGMR analyses, the denominators of the causal estimates were estimated as the effect of the GRS on UK Biobank participants’ own sex-specific Z-scores of BMI, multiplied by 0.5 to reflect the separation by one generation^34–36^ and by the SD of BMI among UK Biobank participants of the sex corresponding to the parent. This gave estimates of parental BMI (kg/m^2^) per unit of the offspring GRS, estimated using models that were unadjusted and adjusted as described above (further details in Supplementary methods, available as Supplementary data at *IJE* online).

The ratio method for IV estimation assumes independence between the numerator and the denominator, which is only assured when they are estimated in different samples. This was the case for the OAI analysis but not for the GRS-PGMR method. Use of the same sample for numerator and denominator also risks over-fitting. Two-sample summary-level PGMR (hereafter; ‘summary-PGMR’) analyses were therefore performed in which IV estimates were made for each of the 97 SNPs using two independent samples and combined using inverse-variance weighting. Denominators for these single-SNP estimates were the effect estimates per BMI-increasing allele reported in the ‘most significant analysis’ from GIANT converted to units of kg/m^2^ using the sex-specific SD of BMI as estimated among UK Biobank participants and halved to reflect the generation gap between instrument and exposure (see Supplementary methods, available as Supplementary data at *IJE* online). Numerators for these single-SNP estimates were obtained using individual-level data from UK Biobank. By convention, the inverse-variance weighted (IVW) mean of these single-SNP estimates is referred to as the ‘IVW estimate’.

Standard errors of the natural logarithm of each IV hazard ratio were reported to indicate their precision. Bias component plots were used to investigate the relative bias of the OAI and GRS-PGMR methods due to various measured covariates.^37^^,^^38^ All analyses were performed in Stata 16.1 and code is provided in the Supplementary material (available as Supplementary data at *IJE* online).

### Sensitivity analyses

In addition to the weighted mean (‘IVW estimate’), single-SNP estimates from summary-PGMR were also combined using the MR–Egger, weighted median and weighted mode methods, each of which is robust to different violations of the assumption of no horizontal pleiotropy—an important violation of MR assumptions.^14^^,^^39–41^

The GRS-PGMR and the summary-PGMR differ in two-respects: the use of independent samples in the IV numerator and denominator and the process by which the PGMR estimate was made. To separate the impact of these two differences, a sensitivity analysis was conducted repeating the summary-PGMR but with both the numerator and denominator estimated in UK Biobank (rather than taking the denominator from GIANT). This approach, which also allowed full adjustment in the denominator, is referred to as ‘summary-PGMR using UK Biobank’.

In further sensitivity analyses, the GRS-PGMR and summary-PGMR analyses were conducted using only the 77 SNPs that reached a genome-wide significance threshold^31^ in GIANT participants of White European ancestry (combined sexes) and the corresponding effect estimates within this subset.

Parents entered follow-up at the birth of their offspring (the parent’s death before this would have prevented their inclusion in the analysis since the offspring would never have been born). Parents’ age at this time could be calculated for parents still living when their offspring participated in UK Biobank but not for those who had died. In the main analysis, we therefore calculated the mean age at parenthood among living parents and used it as the entry to follow-up for those parents who had died. To check sensitivity to this approximation, we repeated the analyses with entry to follow-up set at (i) the 5th and (ii) the 95th percentile of age at parenthood among surviving parents.

Proportionality of hazards was tested in the numerators of the OAI and GRS-PGMR models by correlating the Schoenfeld residuals for BMI with the natural logarithm of analysis time. Departures from proportional hazards were quantified by calculating separate hazard ratios (assuming constant denominators) before and after the age of 70 years.

## Results

BMI was higher in UK Biobank participants with male sex, lower income, lower physical activity, greater age, less education and mothers who smoked at the time of their birth. It was lower in never smokers and higher in ex-smokers. Current alcohol drinkers and retired people were over-represented in the middle quintiles of BMI, whereas other drinking and employment categories were over-represented at the extremes (Table 1).

**Table 1:** Association between offspring body mass index (BMI) and covariables

	Mean (SD) or percentage of variable in BMI quintile					Regression^a^ of BMI on variable	
Variable	1	2	3	4	5	Estimate (95% CI)	*P*-value
BMI (kg/m^2^)^b^	21.7 (1.3)	24.5 (0.6)	26.5 (0.6)	28.8 (0.8)	34.2 (3.8)		
GRS^b^	87.5 (6.2)	88.0 (6.2)	88.5 (6.2)	88.9 (6.2)	89.8 (6.2)	0.10 (0.10, 0.10)	<0.001
Male sex^c^	30.2%	46.1%	55.6%	59.0%	49.8%	0.91 (0.87, 0.94)	<0.001
Average annual pre-tax household income^d^						–0.31 (–0.32, –0.29)	<0.001
<£18 000 (1)	14.9%	13.7%	14.2%	15.5%	19.3%		
£18 000 to £30 999 (2)	23.6%	24.6%	24.8%	25.3%	26.4%		
£31 000 to £51 999 (3)	28.4%	29.1%	29.5%	29.2%	28.7%		
£52 000 to £100 000 (4)	25.3%	25.5%	24.9%	23.8%	21.0%		
>£100 000 (5)	7.8%	7.1%	6.5%	6.2%	4.5%		
Days of moderate physical activity per week^b^	3.81 (2.31)	3.67 (2.27)	3.55 (2.27)	3.43 (2.28)	3.14 (2.31)	–0.22 (–0.23, –0.21)	<0.001
Smoking status^d^						0.32 (0.29, 0.35)	<0.001
Never (0)	62.8%	59.5%	56.5%	53.2%	52.5%		
Former (1)	27.8%	31.7%	34.9%	37.6%	38.8%		
Current (2)	9.5%	8.7%	8.6%	9.2%	8.7%		
Alcohol drinker status^d^						–0.33 (–0.38, –0.27)	<0.001
Never (0)	2.7%	2.1%	1.9%	2.1%	3.0%		
Former (1)	2.9%	2.4%	2.4%	2.7%	3.6%		
Current (2)	94.4%	95.5%	95.7%	95.3%	93.4%		
Age at assessment centre (years)^b^	54.4 (8.1)	55.6 (8.1)	56.1 (8.0)	56.2 (8.0)	55.7 (7.8)	0.02 (0.02, 0.02)	<0.001
Highest education level^e^							<0.001
College or university degree	49.3%	44.5%	40.9%	37.2%	32.5%	reference	
A-levels/AS-levels or equivalent	14.6%	13.7%	13.9%	13.7%	14.0%	0.60 (0.54, 0.66)	<0.001
O-levels/GCSEs or equivalent	22.1%	24.5%	25.7%	26.7%	28.9%	1.05 (1.00, 1.09)	<0.001
CSEs or equivalent	4.6%	5.5%	5.9%	6.8%	7.9%	1.48 (1.40, 1.56)	<0.001
NVQ or equivalent	4.6%	6.4%	8.0%	9.3%	9.9%	1.67 (1.60, 1.75)	<0.001
Other professional qualifications	4.8%	5.4%	5.7%	6.3%	6.8%	1.15 (1.06, 1.23)	<0.001
Current employment status^d^						0.13 (0.10, 0.16)	<0.001
In paid employment (1)	66.4%	64.6%	63.8%	63.8%	64.1%		
Retired (2)	26.1%	30.1%	31.3%	31.0%	28.3%		
Not in paid employment (3)	7.5%	5.3%	5.0%	5.1%	7.6%		
Mother smoked at time of offspring birth^c^	24.8%	27.3%	29.1%	31.6%	35.8%	0.85 (0.81, 0.89)	<0.001
Mother's age at offspring assessment (years)^f^	77.8 (7.9)	78.3 (8.1)	78.3 (8.1)	78.2 (8.1)	77.8 (7.9)		
Father's age at offspring assessment (years)^f^	77.6 (7.2)	77.9 (7.2)	77.9 (7.3)	77.6 (7.3)	77.4 (7.1)		
Mother's age at death^f^ (years)	74.9 (13.2)	74.9 (13.2)	75.0 (13.0)	74.4 (13.0)	73.5 (13.0)		
Father's age at death^f^ (years)	71.8 (12.8)	71.4 (12.9)	71.0 (12.9)	70.7 (12.8)	69.8 (12.8)		

BMI, body mass index; GRS, genetic risk score for BMI; GCSE, general certificate of secondary education; CSE, certificate of secondary education; NVQ, national vocational qualification. See Table 4 for sample sizes. Offspring are UK Biobank participants.

a Separate unadjusted linear regression for each variable with BMI (kg/m^2^) as the outcome.

b Continuous variables. Effect estimates (and corresponding *P-*values) represent the change in BMI (kg/m^2^) per unit increase of variable.

c Binary variables. Effect estimates are for the indicated category relative to the reference category.

d Ordered categorical variables treated as continuous to obtain a single *P-*value and effect estimate per category increase in the variable. Values in brackets were assigned to each category.

e Categorical variable providing effect sizes relative to the reference category (college or university degree). *P-*values are for each comparison with the reference category and overall, from a likelihood ratio test comparing models with and without the categorical variable.

f The association of these variables with BMI was not estimated because samples available were not representative of the data set.

Covariate associations with the GRS were considerably weaker but non-zero, mostly in the same direction as the BMI associations (Table 2). The GRS was higher in those with male sex, lower income, less education and mothers who smoked at the time of offspring birth. It was also higher in current and former smokers and in never and former alcohol drinkers.

**Table 2:** Association of the offspring genetic risk score (GRS) for body mass index (BMI) with covariables

	Mean (SD) or percentage of variable in GRS quintile					Regression^a^ of GRS on variable	
Variable	1	2	3	4	5	Estimate (95% CI)	*P*-value
BMI (kg/m^2^)^b^	26.3 (4.2)	26.8 (4.4)	27.1 (4.6)	27.4 (4.7)	28.0 (5.0)	0.18 (0.18, 0.19)	<0.001
GRS^b^	79.8 (2.9)	85.2 (1.1)	88.5 (0.9)	91.8 (1.1)	97.3 (2.9)		
Male sex^c^	47.6%	48.2%	48.3%	48.1%	48.5%	0.07 (0.02, 0.12)	0.007
Average annual pre-tax household income^d^						–0.06 (–0.08, –0.04)	<0.001
<£18 000 (1)	15.3%	15.3%	15.5%	15.7%	15.9%		
£18 000 to £30 999 (2)	24.6%	24.9%	25.0%	25.1%	25.2%		
£31 000 to £51 999 (3)	29.1%	28.7%	29.1%	29.0%	29.1%		
£52 000 to £100 000 (4)	24.3%	24.7%	24.2%	23.8%	23.6%		
>£100 000 (5)	6.7%	6.4%	6.2%	6.4%	6.2%		
Days of moderate physical activity per week^b^	3.52 (2.30)	3.53 (2.30)	3.51 (2.31)	3.53 (2.30)	3.51 (2.30)	–0.01 (–0.02, 0.01)	0.329
Smoking status^d^						0.15 (0.11, 0.19)	<0.001
Never (0)	57.7%	57.8%	56.6%	56.5%	55.9%		
Former (1)	33.6%	33.6%	34.4%	34.4%	34.8%		
Current (2)	8.6%	8.6%	9.0%	9.1%	9.3%		
Alcohol drinker status^d^						–0.10 (–0.18, –0.03)	0.008
Never (0)	2.3%	2.3%	2.3%	2.4%	2.4%		
Former (1)	2.7%	2.8%	2.7%	2.9%	2.9%		
Current (2)	95.0%	94.9%	94.9%	94.7%	94.7%		
Age at assessment centre (years)^b^	55.6 (8.0)	55.6 (8.0)	55.6 (8.0)	55.6 (8.0)	55.5 (8.0)	0.00 (–0.01, 0.00)	0.011
Highest education level^e^							<0.001
College or university degree	41.5%	41.2%	41.2%	40.2%	40.2%	reference	
A-levels/AS-levels or equivalent	14.3%	14.0%	14.0%	14.2%	13.6%	0.01 (–0.07, 0.09)	0.816
O-levels/GCSEs or equivalent	25.3%	25.6%	25.3%	25.6%	26.0%	0.13 (0.07, 0.20)	<0.001
CSEs or equivalent	5.8%	6.0%	5.9%	6.3%	6.5%	0.31 (0.20, 0.42)	<0.001
NVQ or equivalent	7.3%	7.6%	7.7%	7.8%	7.8%	0.25 (0.15, 0.35)	<0.001
Other professional qualifications	5.7%	5.6%	5.9%	5.9%	5.9%	0.16 (0.05, 0.28)	0.004
Current employment status^d^						–0.02 (–0.06, 0.02)	0.320
In paid employment (1)	64.4%	64.6%	64.5%	64.5%	64.7%		
Retired (2)	29.4%	29.3%	29.4%	29.3%	29.3%		
Not in paid employment (3)	6.2%	6.1%	6.1%	6.2%	6.0%		
Mother smoked at time of offspring birth^c^	29.2%	29.7%	29.1%	30.1%	30.3%	0.14 (0.08, 0.19)	<0.001
Mother's age at offspring assessment (years)^f^	78.2 (8.0)	78.2 (8.0)	78.1 (8.0)	78.0 (8.1)	78.0 (8.0)		
Father's age at offspring assessment (years)^f^	77.7 (7.2)	77.8 (7.2)	77.8 (7.2)	77.6 (7.3)	77.5 (7.2)		
Mother's age at death^f^ (years)	74.7 (13.1)	74.5 (13.0)	74.6 (13.1)	74.5 (13.1)	74.2 (13.1)		
Father's age at death^f^ (years)	71.2 (12.8)	71.0 (12.9)	70.9 (12.8)	70.9 (12.8)	70.7 (12.9)		

BMI, body mass index; GRS, genetic risk score for BMI; GCSE, general certificate of secondary education; CSE, certificate of secondary education; NVQ, national vocational qualification. See Table 4 for sample sizes. Offspring are UK Biobank participants. BMI-SNP associations to construct the GRS were taken for the 97 single-nucleotide polymorphisms (SNPs) identified as genome-wide significant at *P* < 5 × 10^–8^ in the ‘most significant analysis’ of Locke *et al.* 2015.^31^

a Separate linear regression for each variable with GRS as the outcome, adjusted for the first 10 genetic principal components.

b Continuous variables. Effect estimates (and corresponding *P-*values) represent the change in GRS per unit increase of variable.

c Binary variables. Effect estimates are for the indicated category relative to the reference category.

d Ordered categorical variables treated as continuous to obtain a single *P-*value and effect estimate per category increase in the variable. Values in brackets were assigned to each category.

e Categorical variable providing effect sizes relative to the reference category (college or university degree). *P*-values are for each comparison with the reference category and overall, from a likelihood ratio test comparing models with and without the categorical variable.

f The association of these variables with GRS was not estimated because samples available were not representative of the data set.

Parental mortality was associated with similar factors to offspring BMI except that mortality was lower among parents of older offspring and was not associated with offspring physical activity (Table 3).

**Table 3:** Association between offspring covariables and parental all-cause mortality

	Mothers		Fathers	
Variable	HR (95% CI)^a^	*P*-value	HR (95% CI)^a^	*P*-value
BMI (kg/m^2^)^b^	1.02 (1.02, 1.02)	<0.001	1.02 (1.02, 1.02)	<0.001
GRS^b^	1.00 (1.00, 1.00)	<0.001	1.00 (1.00, 1.00)	<0.001
Male sex^c^	1.02 (1.01, 1.03)	<0.001	1.01 (1.00, 1.02)	0.208
Average annual pre-tax household income^d^	0.93 (0.93, 0.94)	<0.001	0.94 (0.93, 0.94)	<0.001
Days of moderate physical activity per week^b^	1.00 (1.00, 1.00)	0.300	1.00 (1.00, 1.00)	0.521
Smoking status^d^	1.03 (1.02, 1.04)	<0.001	1.05 (1.05, 1.06)	<0.001
Alcohol drinker status^d^	0.97 (0.96, 0.99)	<0.001	0.98 (0.96, 0.99)	<0.001
Age at assessment centre (years)^b^	0.98 (0.98, 0.99)	<0.001	0.98 (0.97, 0.98)	<0.001
Highest education level^e^		<0.001		<0.001
College or university degree	Reference		Reference	
A-levels/AS-levels or equivalent	1.08 (1.06, 1.10)	<0.001	1.08 (1.06, 1.09)	<0.001
O-levels/GCSEs or equivalent	1.16 (1.14, 1.18)	<0.001	1.18 (1.17, 1.20)	<0.001
CSEs or equivalent	1.30 (1.27, 1.34)	<0.001	1.30 (1.27, 1.33)	<0.001
NVQ or equivalent	1.28 (1.25, 1.30)	<0.001	1.28 (1.25, 1.30)	<0.001
Other professional qualifications	1.15 (1.13, 1.18)	<0.001	1.16 (1.14, 1.18)	<0.001
Current employment status^d^	1.03 (1.02, 1.04)	<0.001	1.01 (1.00, 1.02)	0.003
Mother smoked at time of offspring birth^c^	1.70 (1.68, 1.72)	<0.001	1.25 (1.23, 1.26)	<0.001

HR, hazard ratio; BMI, body mass index; GRS, genetic risk score for BMI; GCSE, general certificate of secondary education; CSE, certificate of secondary education; NVQ, national vocational qualification. *N* = 231 637 mothers and 227 880 fathers of 233 361 UK Biobank participants.

a HR were from separate Cox proportional hazards models with age as the time axis and adjusted for secular trends (date of birth).

b Continuous variables. Hazard ratios (and corresponding *P-*values) are per unit increase of variable.

c Binary variables. Effect estimates are for the indicated category relative to the reference category.

d Ordered categorical variables treated as continuous to obtain a single *P-*value and HR per category increase in the variable. Categories were assigned values as described in Tables 1 and 2.

e Categorical variable providing hazard ratios relative to the reference category (college or university degree). *P-*values are for each comparison with the reference category and overall, from a likelihood ratio test comparing models with and without the categorical variable.

Data were available for 120 489 mother–daughter (MD) pairs, 111 148 mother–son (MS) pairs, 118 181 father–daughter (FD) pairs and 109 699 father–son (FS) pairs (Figure 2). These comprised 121 044 unique daughters and 112 317 unique sons (Table 4). The mean BMI (SD) was 26.69 kg/m^2^ (5.03) for daughters and 27.59 kg/m^2^ (4.10) for sons. At the time of offspring UK Biobank participation, 127 938 mothers (55%) and 165 769 fathers (73%) were deceased.

**Table 4:** Descriptive characteristics by offspring sex

	Sample size		Mean (SD) or percentage	
Variable	Daughters	Sons	Daughters	Sons
BMI (kg/m^2^)	121 044	112 317	26.7 (5.0)	27.6 (4.1)
GRS	121 044	112 317	88.5 (6.2)	88.6 (6.2)
Male sex	121 044	112 317	0.0%	100.0%
Average annual pre-tax household income	121 044	112 317		
<£18 000	21 675	14 566	17.9%	13.0%
£18 000 to £30 999	31 766	26 455	26.2%	23.6%
£31 000 to £51 999	34 102	33 557	28.2%	29.9%
£52 000 to £100 000	26 650	29 640	22.0%	26.4%
>£100 000	6851	8099	5.7%	7.2%
Days of moderate physical activity per week	121 044	112 317	3.55 (2.31)	3.48 (2.29)
Smoking status	121 044	112 317		
Never	74 023	58 779	61.2%	52.3%
Former	37 841	41 864	31.3%	37.3%
Current	9180	11 674	7.6%	10.4%
Alcohol drinker status	121 044	112 317		
Never	3921	1563	3.2%	1.4%
Former	3585	2928	3.0%	2.6%
Current	113 538	107 826	93.8%	96.0%
Age at assessment centre (years)	121 044	112 317	55.1 (7.9)	56.1 (8.1)
Highest education level	121 044	112 317		
College or university degree	47 956	47 432	39.6%	42.2%
A-levels/AS-levels or equivalent	18 189	14 462	15.0%	12.9%
O-levels/GCSEs or equivalent	34 083	25 607	28.2%	22.8%
CSEs or equivalent	7333	6 965	6.1%	6.2%
NVQ or equivalent	5755	12 050	4.8%	10.7%
Other professional qualifications	7728	5 801	6.4%	5.2%
Current employment status	121 044	112 317		
In paid employment	76 445	74 170	63.2%	66.0%
Retired	35 468	33 020	29.3%	29.4%
Not in paid employment	9131	5127	7.5%	4.6%
Mother smoked at time of offspring birth	107 983	98 672	29.1%	30.4%
Mother's age at offspring assessment (years)	55 997	47 895	78.0 (7.9)	78.2 (8.2)
Father's age at offspring assessment (years)	34 000	28 813	77.6 (7.1)	77.8 (7.3)
Mother's age at death (years)	64 583	63 355	74.3 (13.2)	74.8 (12.9)
Father's age at death (years)	84 494	81 275	70.9 (12.8)	71.0 (12.9)

BMI, body mass index; GRS, genetic risk score for BMI; GCSE, general certificate of education; CSE, certificate of secondary education; NVQ, national vocational qualification. Offspring are UK Biobank participants.

Differences in parental BMI per kg/m^2^ of offspring BMI and per unit of the offspring GRS, used as denominators in the OAI and GRS-PGMR analyses, respectively, are shown in Table 5. The differences per kg/m^2^ of offspring BMI that we chose as OAI denominators on the basis of the demographic similarity of the study sample^33^ to UK Biobank were rather smaller than other studies of similarly aged offspring, especially for the FD association (Supplementary Table S3, available as Supplementary data at *IJE* online).

**Table 5:** Associations of body mass index (BMI) with offspring BMI and offspring genetic risk score (GRS) instruments in the offspring as instrument (OAI) and proxy-genotype Mendelian randomization (PGMR) analyses, respectively

Parent	Participants	Adjustment	*N*	Mean difference (95% CI)^a^	*P*-value	*R* ^2^ (%)^b^	F^c^
Difference in parental BMI (kg/m^2^) per kg/m^2^ of offspring BMI (denominator for OAI)							
Mothers	Daughters	Unadjusted^d^	4696	0.168 (0.144, 0.192)	<0.001	5.87%	188.2
Mothers	Daughters	Adjusted^e^	4696	0.139 (0.118, 0.161)	<0.001	4.02%	160.6
Mothers	Sons	Unadjusted^d^	4651	0.192 (0.164, 0.220)	<0.001	4.60%	180.6
Mothers	Sons	Adjusted^e^	4651	0.168 (0.140, 0.196)	<0.001	3.53%	138.3
Fathers	Daughter	Unadjusted^d^	4696	0.079 (0.061, 0.097)	<0.001	2.13%	74.0
Fathers	Daughter	Adjusted^e^	4696	0.064 (0.048, 0.080)	<0.001	1.40%	61.5
Fathers	Sons	Unadjusted^d^	4651	0.136 (0.114, 0.158)	<0.001	3.80%	146.8
Fathers	Sons	Adjusted^e^	4651	0.132 (0.113, 0.152)	<0.001	3.58%	176.0

Difference in parental BMI (kg/m^2^) per unit of offspring GRS (denominator for GRS-PGMR)							
Mothers	Both	Unadjusted^d^	233 361	0.055 (0.052, 0.058)	<0.001	1.85%	4418.1
Mothers	Both	Adjusted^e^	233 361	0.054 (0.051, 0.057)	<0.001	1.77%	4376.7
Fathers	Both	Unadjusted^d^	233 361	0.045 (0.042, 0.047)	<0.001	1.85%	4418.1
Fathers	Both	Adjusted^e^	233 361	0.044 (0.041, 0.046)	<0.001	1.77%	4376.7

BMI, body mass index; GRS, genetic risk score for BMI; PGMR, proxy-genotype Mendelian randomization. Intergenerational BMI associations were adapted from estimates made using 1958 British Birth Cohort data.^24^ The genetic variants comprising the GRS were weighted by the BMI-SNP associations for the 97 single-nucleotide polymorphisms (SNPs) identified at a genome-wide significance threshold of *P* < 5 × 10^–8^ in the ‘most significant analysis’ of Locke *et al*. 2015^31^ using GIANT data. The initial regression of BMI on GRS in UK Biobank was conducted in combined sexes using sex-specific Z-scores of BMI. The resulting coefficient was converted to kg/m^2^ using the standard deviation of BMI appropriate to the sex of the parent (See Supplementary methods, available as Supplementary data at *IJE* online for details).

a Linear regression coefficients for parental BMI (kg/m^2^) per kg/m^2^ of offspring BMI (OAI) or per BMI-increasing allele of offspring GRS (PGMR).

b Partial *R*^2^ reflecting the proportion of BMI variance explained by the instrument.

c Partial F-statistic measuring the strength of the instrument.

d Unadjusted analyses in UK Biobank data were adjusted only for offspring date of birth and, in genetic analyses only, for the first 10 genetic principal components. The closest available adjustment set was used for intergenerational BMI associations.

e Adjusted analyses were additionally adjusted for highest level of education attained, current employment status, smoking status, alcohol intake, physical activity, age when attended assessment centre and average household income before tax. The closest available adjustment set was used for intergenerational BMI associations.

OAI analyses provided evidence that higher BMI increased mortality among parents, with adjusted hazard ratios (HRs) per kg/m^2^ higher BMI ranging from 1.08 (95% CI: 1.06, 1.10) for mothers using their son’s BMI as the instrument to 1.23 (95% CI: 1.16, 1.29) for fathers using their daughter’s BMI as the instrument (Table 6). Estimates were marginally changed by adjustment but were rather higher when BMI of daughters, rather than sons, was used as the instrument.

**Table 6:** Hazard ratios for all-cause mortality per kg/m^2^ of body mass index (BMI) using offspring BMI or offspring genotype as an instrument for parent’s BMI

			Unadjusted model^a^			Adjusted model^b^		
Parent	Participants	*N*	HR (95% CI)	*P*-value	SE[Ln(HR)]	HR (95% CI)	*P*-value	SE[Ln(HR)]
Offspring BMI as instrument (OAI)								
Mothers	Daughters	120 489	1.11 (1.09, 1.13)	<0.001	0.009	1.11 (1.09, 1.14)	<0.001	0.010
Mothers	Sons	111 148	1.08 (1.07, 1.10)	<0.001	0.008	1.08 (1.06, 1.10)	<0.001	0.009
Mothers	Both^c^	231 637	1.10 (1.08, 1.11)	<0.001	0.006	1.10 (1.08, 1.11)	<0.001	0.007
Fathers	Daughters	118 181	1.22 (1.16, 1.28)	<0.001	0.025	1.23 (1.16, 1.29)	<0.001	0.028
Fathers	Sons	109 699	1.14 (1.11, 1.17)	<0.001	0.012	1.12 (1.10, 1.14)	<0.001	0.011
Fathers	Both^c^	227 880	1.16 (1.13, 1.18)	<0.001	0.011	1.13 (1.11, 1.16)	<0.001	0.010
Proxy-genotype Mendelian randomization using genetic risk scores (GRS-PGMR)								
Mothers	Both	231 637	1.03 (1.02, 1.05)	<0.001	0.008	1.03 (1.01, 1.04)	0.001	0.008
Fathers	Both	227 880	1.06 (1.04, 1.07)	<0.001	0.009	1.05 (1.03, 1.07)	<0.001	0.009
IVW estimates from summary-level proxy-genotype Mendelian randomization with independent samples (summary-PGMR)								
Mothers	Both	231 637	1.03 (1.01, 1.04)	<0.001	0.007	1.02 (1.01, 1.04)	0.001	0.007
Fathers	Both	227 880	1.05 (1.03, 1.06)	<0.001	0.007	1.04 (1.02, 1.05)	<0.001	0.007

BMI, body mass index (kg/m^2^); HR, hazard ratio; SE, standard error; PGMR, proxy-genotype Mendelian randomization.

a Unadjusted PGMR analyses were adjusted for the first 10 genetic principal components and for secular trends (offspring date of birth). Unadjusted OAI analyses were adjusted for secular trends only.

b Adjusted analyses were additionally adjusted for highest level of education attained, current employment status, smoking status, alcohol intake, physical activity, age when attended assessment centre and average household income before tax.

c Estimates from sons and daughters were combined in meta-analyses, which indicated substantial heterogeneity between them (*I*^2^ = 77.6, *P*_heterogeneity_ = 0.035 for mothers, *I*^2^ = 83.2, *P*_heterogeneity_ = 0.015 for fathers).

PGMR analyses also suggested that higher BMI increased mortality risk, but estimates were closer to the null than those from OAI analyses. Adjusted HRs per kg/m^2^ higher BMI were 1.02 (95% CI: 1.01, 1.04) for mothers and 1.04 (95% CI: 1.02, 1.05) for fathers in the IVW estimates from summary-PGMR analyses (Table 6). Results for GRS-PGMR were very similar to these and adjustment slightly attenuated the estimates.

**Figure 3:**
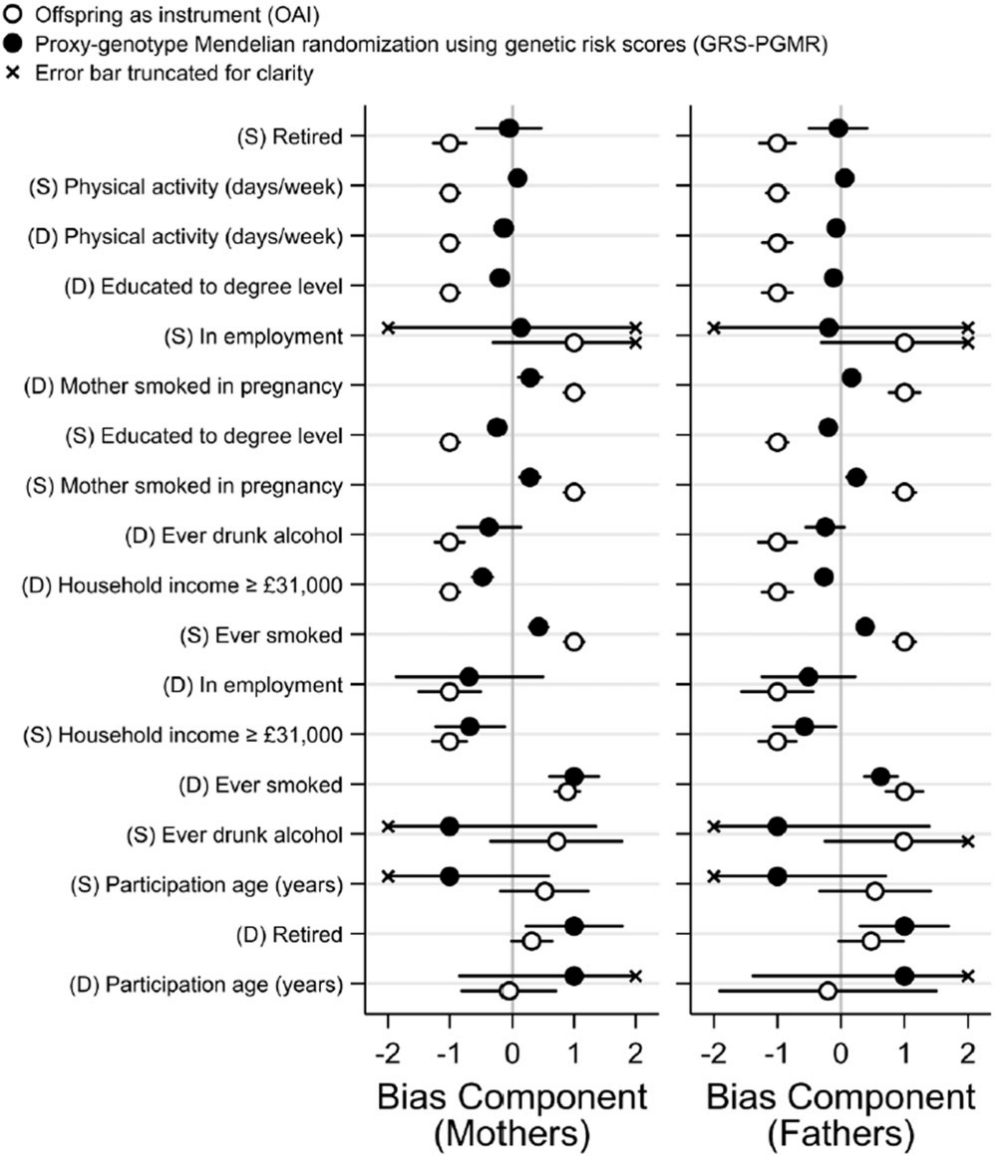
Bias components from measured covariates. Separate analyses were made in the data available for mothers and fathers, and for sons (S) and daughters (D). Bias components are on an arbitrary, relative scale and are comparable between the two instrumental variable (IV) methods for each covariate but not between covariates or sexes. They were therefore scaled for plotting by the absolute magnitude of the larger of each pair for ease of presentation. Plotted bias components are ordered by absolute relative bias [bias in offspring as instrument (OAI)/bias in genetic risk score proxy-genotype Mendelian randomization (GRS-PGMR)]. Error bars are 95% CIs

The OAI estimates for maternal mortality with sons and daughters combined were slightly more precise than the corresponding PGMR estimates with the same sample size. In contrast, those for paternal mortality were slightly less precise than the corresponding PGMR estimates, due to the low precision of the estimate using daughters and the greater heterogeneity between the two estimates. Bias components for measured covariates were mostly greater for the OAI analysis than for the GRS-PGMR analysis and where this pattern was reversed, the magnitudes of the bias components could not be confidently distinguished (Figure 3).

### Sensitivity analyses

MR–Egger intercepts were close to the null, suggesting that any unbalanced horizontal pleiotropy was minimal. Pleiotropy-adjusted results were marginally attenuated compared with the IVW estimate, with considerably reduced precision. The summary-PGMR using UK Biobank gave almost identical results to the more conventional summary-PGMR using independent samples (Supplementary Table S4, available as Supplementary data at *IJE* online). Sensitivity analyses with alternative SNP selection or entry to follow-up gave analogous results to the main analysis (Supplementary Tables S5 and S6, available as Supplementary data at *IJE* online). In OAI and GRS-PGMR analyses, there was strong evidence of non-proportional hazards. BMI was positively associated with mortality both before and after the age of 70 years, but HRs were attenuated in the older age group (Supplementary Table S7, available as Supplementary data at *IJE* online).

## Discussion

In this intergenerational study of European-ancestry British individuals, results from both OAI and PGMR analyses supported a causal role of higher BMI increasing mortality. However, causal estimates from OAI analyses were considerably greater in magnitude than those from PGMR analyses, dependent on the parent–offspring sex-specific combination. The magnitude and direction of these results do not have a clear causal interpretation, but the PGMR estimates were similar in magnitude to published conventional MR estimates such as those from a recent analysis in UK Biobank,^32^ which found a 3% increase in the hazard of all-cause mortality per kg/m^2^ higher BMI. Previous estimates using the OAI method^12^^,^^15^ have been >3% but estimates lie within the PGMR range found here. The OAI estimates presented here are higher than previous OAI estimates as well as the MR estimates presented here and elsewhere. They are also variable according to the sex of the parent and offspring whose BMI was used as an instrument. Note that results presented here for mothers and fathers are not independent of each other, since they are derived using the BMI or genotype of a largely overlapping set of offspring. BMI is a complex measure of body composition. It is unable to differentiate between fat mass and lean mass and is not an appropriate measure in pregnancy. Therefore, being classified as overweight or obese may appear protective against mortality.^42^ A possible direction for future studies may include implementing these approaches using alternative measures such as the waist-to-hip ratio.^43^

If two instruments applied to similar samples are both valid, then they should give similar estimates of the effect of the exposure on the outcome.^44^ Getting different estimates using son’s or daughter’s BMI as an instrument in the current study indicates that at least one of the instruments used may violate the assumptions for a valid instrument. The heterogeneity also means that the meta-analysed results for combined sons and daughters should be treated with caution.

There is much discussion in the literature regarding the non-linear effect of BMI on mortality.^32^^,^^45^^,^^46^ The focus of our analysis was to compare linear estimates from two different IV methods. Non-linear IV methods to investigate the shape of the BMI–mortality relationship require individual-level data from a single sample, so could not be applied using our two-sample OAI and summary-PGMR approaches. We highlight this as an area for future development.

Valid IVs are not associated with factors confounding the relationship between the exposure and outcome and IVs are invalidated if those factors also affect the instrument. However, bias can be accounted for if the factor confounding the instrument and outcome is accurately measured and appropriately adjusted for.^21^ The choice of adjustment variables made little difference to estimates made by the OAI or PGMR methods, suggesting that none of the measured covariates substantially invalidates either instrument. Adjusting for covariates would violate MR assumptions if they were in fact colliders, not confounders, in the relationship between BMI and mortality. We argue that the behavioural and socio-economic covariates adjusted for here are more likely confounders and adjustment for them had little effect on the estimates anyway.

We were also unable to adjust for pre-existing disease. This may be an important confounder of associations between BMI and mortality but, like other confounders, is accounted for by IV methods because disease in the parents is unlikely to affect offspring BMI or genetic variation. We note, however, that the present OAI analysis may be less robust to reverse causation than previous OAI studies in which the instrument was measured in younger offspring,^10–12^ if caring responsibilities for frail parents affect the BMI of middle-aged offspring. All confounder variables were measured in the offspring and are thus proxy measures in which some residual confounding may remain. There may additionally be unmeasured confounders in the offspring for which we are unable to adjust.

Our results were largely consistent when applying alternative methods to the IVW, such as the weighted median and weighted mode. Estimates obtained through the weighted median are robust to pleiotropic bias when a weighted half of the SNPs in the estimation are not pleiotropic.^47^ Similarly, the weighted mode approach is consistent when the largest (weighted) set of SNPs, grouped by the per SNP casual effect estimates, are valid.^48^ Estimates obtained via these weighted methods may be more appropriate, although, in the presence of directional pleiotropy across all the SNPs, may still give biased causal effect estimates.^40^^,^^47^ We therefore also applied MR–Egger to test for directional pleiotropy across all SNPs. MR–Egger estimates are robust to pleiotropy under the assumption that the pleiotropic effects are uncorrelated with the SNP–exposure association. Each of these approaches gave similar effect estimates. We note however that the weighted mode and MR–Egger approaches can have low power to detect causal effect. Estimates obtained from these approaches were in line with other methods, although less precise.

UK Biobank is a large-scale population-based study with rich, intergenerational data. Nonetheless, UK Biobank participants have better health, more education and a more favourable socio-economic position than the general UK population.^49^ If the effect of BMI on survival is more pronounced at higher BMI, the results presented here are underestimated. Participants are also a healthy subset of their birth cohort because they have necessarily survived to middle age; this good health may be shared by their parents. Although the cohort was large, the use of a complete-case analysis and the necessary exclusions to eliminate genetic confounding reduced the sample size and made it less representative, limiting the generalizability of these results to a wider population. Particularly, recent work has shown that there are sex differentials in the relationship between BMI and participation in UK Biobank with BMI-increasing variants at the well-characterized *FTO* locus being more frequently observed in male participants.^50^ This differential participation could explain the sex differences in estimated effects we observed if differences in participation also depend on any other factors that affect mortality.

Missing data on parental age at offspring’s birth for deceased parents necessitated their estimation from surviving parents. Substantial bias from this is unlikely because parenthood generally occurs within a relatively narrow age band in the context of a person’s lifespan, at which mortality is unusual. Furthermore, little sensitivity was demonstrated to this approximation of parental age. In the absence of IGA within UK Biobank, we used values from the literature for people of a similar age and genetic background. Use of independent samples for the associations between the instrument and exposure and between instrument and outcome used in the IV estimation can improve power and avoid bias due to winners’ curse and weak instruments but, as discussed below, it can also add bias if there is heterogeneity between the samples. Both IV methods require several assumptions: (i) the IV (here, offspring BMI, GRS or individual SNPs) is associated with the exposure of interest (here, parental BMI), (ii) there is no relationship between the IV and the outcome (here, parental mortality) except through the exposure and (iii) there are no confounders of the instrument and outcome.^8^ Interpretation of the effect estimates as average causal effects also requires (iv) monotonicity between the exposure and the unmeasured causal instrument, which is a common cause of the measured instrument and the exposure.^7^^,^^51^ The validity of the first assumption for OAI and PGMR methods is clearly demonstrated here and in the literature.^31–33^^,^^52^^,^^53^ Some examination of the second assumption in PGMR can be made using MR–Egger, although this makes several strong assumptions of its own.^40^ However, our estimated MR–Egger intercept indicated little evidence of directional pleiotropy. The bias component plots test the third assumption for the measured covariates, but only in relative terms comparing alternative methods; they cannot estimate the absolute magnitude of the bias that would result from the omission of these measured covariates. They indicate that bias in OAI and PGMR analyses would usually have been in the same direction, with the bias being relatively greater for OAI than for PGMR. For the fourth assumption, we argue that a monotone effect of parental genotype (the underlying causal instrument in a PGMR analysis) on parental BMI is likely. The genetic variants included in the PGMR analysis form a relatively small component of the genetic component of the underlying causal instrument for the OAI analysis, together with environmental factors common to parent and offspring. We cannot be so confident that this latter component has a monotone association with BMI. Here, the assumption of monotonicity implies an interpretation of the estimated effect as a weighted average causal effect with undefined weights among the population.^7^^,^^51^ The estimated IV effects may therefore differ from the unweighted average causal effect if the effect size varies according to the exposure (i.e. non-linearity) or by covariates (some of which we have adjusted for). The finding of non-proportional hazards indicates that it is also an ill-defined combination of differing associations at different ages. This may be due to differential selection by unmeasured covariates.^54^ It is notable, though, that HRs were in the same direction in the two main age groups examined here. Furthermore, BMI differs over the life course, so its association with either instrument (and thus the IV estimate of its effect on an outcome) may vary according to when the BMI is measured. It has been suggested^23^ that an IV effect estimate for a time-varying exposure should be interpreted as an effect of a change in the underlying lifelong liability to that exposure, as the association of the IV with the exposure at other time points will form part of the overall effect estimated.

Overall, these assumptions severely constrain the quantitative interpretation that may be placed upon the estimated HRs. Nonetheless, they are likely to apply similarly to each of the analyses presented here, allowing the comparison of estimates between them. Any two-sample IV analysis assumes that the instrument–exposure association is similar in the two samples. The similarity between GRS-PGMR and summary-PGMR analyses is reassuring in this respect, but violation of this assumption may have influenced the OAI estimates. The variability of OAI estimates for different combinations of parents and offspring, and their departure from previous OAI estimates, appears to originate in the denominators (intergenerational BMI associations; Table 5). These were rather smaller in the 1958 British birth cohort we used^33^ than in comparable cohorts (Supplementary Table S3, available as Supplementary data at *IJE* online). Previous studies have found that mean differences in kg/m^2^ units are strongest for MS and weakest for FD pairs. Although there may be biological reasons for this, it also expected from the greater variability in BMI generally found among women. Differences between the sexes and generations in the standard deviation of BMI were particularly pronounced in the 1958 British birth cohort.

A further assumption in a PGMR analysis is that the IGA between the parental exposure and offspring instrument is half the association within an individual, since parents and offspring are 50% related.^36^ The standard error of the IGA is further assumed to be equal to that found within an individual. This assumption depends on the genetic associations with BMI remaining similar in successive generations, but there is evidence that they have increased.^55^ This would result in the overestimation of associations between the instrument and exposure, thus underestimating the average causal effect of BMI in the PGMR analyses.

Most IVs are associated with the exposure due to a causal effect, but this is not plausible for the instruments used here. If the reverse is true and the exposure causes the instrument, then the instrument is not valid because the exposure is no longer a collider blocking any pathways from the instrument to unmeasured common causes of parental BMI and parental mortality (Figure 1).^56^ We must therefore assume that offspring BMI and genotype are associated with parental BMI because they share common causes, making them proxy instruments. This complicates the causal interpretation of effect estimates.^7^ Parental genetics is a clear common cause of offspring genetics and parental BMI. The strong genetic and environmental influences on BMI shared by parents and offspring suggest that the association between offspring and parental BMI is also driven primarily by common causes. It must further be assumed that these common causes are distinct from the common causes of parental BMI and parental mortality (a special case of the third IV assumption analysis described above). Although the genetic common causes of parental and offspring BMI are unlikely to affect parental mortality (except via parental BMI), it is likely that some of the diverse socio-economic and behavioural environmental factors affecting parental and offspring BMI also affect parental mortality.

Assortative mating can bias estimates from OAI or PGMR analyses, either by single-trait or cross-trait assortative mating.^57^^,^^58^ We cannot investigate this further because we lack parental genotypes. We note, and depict in Figure 1, the possible dynastic effects that may be present in this study.^57^ This may induce confounding between the instrument and outcome in both approaches. However, the bias induced by this is likely equally present in both methods under comparison. The unmediated effect of parental genotype on offspring genotype would likely strengthen offspring genotype as an instrument as both the direct and indirect genetic effects are captured.^58^^,^^59^

This study investigated the underlying causal relationship between BMI and mortality using two methods—OAI and PGMR—and compared statistical power, magnitude of bias, violation of assumptions and overall results. These non-independent methods relate to differently indexed genetic intergenerational associations of BMI. Consistently with the literature, results from both approaches supported an average causal effect of higher BMI on mortality. PGMR analyses were more robust to confounding but may have given conservative estimates if the genetic impact on adiposity increased between generations. The OAI estimates were much greater in magnitude than previous OAI or MR estimates, probably due to violation of the assumption of homogeneity between samples. We propose that these two methods should be used, with due regard to their likely biases, for triangulation of evidence^60–62^ on the causal effects of BMI, in combination with other approaches such as conventional MR, multivariable-adjusted analyses, randomized–controlled trials, natural experiments and the use of early-life BMI (before reverse causation has had a major impact) as an instrument for later BMI.^63^

## Ethics approval

UK Biobank was approved by the Northwest Research Ethics committee (REC reference 11/NW/0382).

## Supplementary Material

dyac159_Supplementary_Data

## Data Availability

UK Biobank data are available upon application as described at https://www.ukbiobank.ac.uk/enable-your-research/apply-for-access.
