# Supplementary material for: Comparison of intergenerational instrumental variable analyses of body mass index and mortality in UK Biobank
Source: Int J Epidemiol. 2022 Aug 10;52(2):545–61. doi: 10.1093/ije/dyac159 (PMC10114047; doi:10.1093/ije/dyac159)
Supplement: dyac159_Supplementary_Data [file dyac159_supplementary_data.zip › dyac159_Supplementary_Data/ije-2021-09-1429-File006.docx]

**Supplementary Methods for:**

**Comparison of intergenerational instrumental variable analyses of BMI and mortality in UK Biobank**

Ciarrah-Jane Barry, David Carslake, Kaitlin H. Wade, Eleanor Sanderson and George Davey Smith

**Classification of covariates**

Month and year of birth were amalgamated into a “Date of birth” variable by adding one twelfth of a year to the year of birth for each complete month such that for example April 1953 became 1953.25. Ethnicity was answered via branching questions, first establishing an ethnic group, then specific subgroups (e.g., “White or White British” led to “British”, “Irish” etc). Principal component analysis of genotypes confirmed defined ethnicity as “White European” or not and genotyping analysis confirmed sex (1). Current employment status was recorded in nine pre-defined categories; 1) in paid employment or self-employed, 2) retired, 3) looking after home and/or family, 4) unable to work because of sickness/disability, 5) unemployed, 6) doing unpaid/voluntary work, 7) full/part-time student, 8) none of the above and 9) prefer not to answer. We combined categories 3-7 into a “not in paid employment” category for all analyses. Categories 8-9 were coded missing. Similarly, highest level of qualification was recorded in eight pre-defined categories; 1) college or university degree, 2) A/AS-levels, 3) O-levels/GCSEs, 4) CSEs, 5) NVQ/HND/HNC, 6) other professional qualifications, 7) none of the above and 8) prefer not to answer. Categories 7-8 were coded missing. Physical activity was recorded as number of days per week on which moderate physical activity was undertaken. Participants reported their smoking status and alcohol drinking as “never”, “former” or “current”. Household income was recorded with a choice of seven levels; 1) less than £18 000, 2) £18 000 to £30 999, 3) £31 000 to £51 999, 4) £52 000 to £100 000, 5) greater than £100 000, 6) do not know and 7) prefer not to answer. Categories 6 and 7 were coded missing.

**Genetic data**

The genome-wide association study of body mass index (BMI) by the Genetic Investigation of ANthropometric Traits (GIANT) consortium (up to 339,334 people) found 97 SNPs which were associated with BMI at P<5x10^-8^ in the “most significant analysis” (2). One of these, rs12016871, was not present in the UK Biobank imputed genetic data, so a proxy SNP (rs4771122) in close linkage disequilibrium (r^2^=0.876, distance=2398bp) was used in its place. Mean differences in BMI per BMI-increasing allele in the most significant analysis were taken for calculation of genetic risk scores (GRS) and for use in the summary-level proxy-genotype Mendelian randomization (summary-PGMR) analyses using independent samples. These mean differences were reported in units of standard deviations (SD) of BMI and did not show any consistent differences between men and women on this scale. We therefore used the same values on this scale for men and women.

To calculate the GRS, genotypes of UK Biobank participants at the 97 loci identified in GIANT were first inverted (subtracted from 2) if they coded for the BMI-decreasing allele. Each genotype (number of BMI-increasing alleles) was then multiplied by its corresponding genetic effect on BMI from GIANT and the products were summed. This sum was divided by the sum of the genetic effects and multiplied by 97 to give a GRS representing the total number of BMI-increasing alleles, weighted by the effect size of each allele. In a sensitivity analysis, we instead used the subset of 77 alleles which were associated with BMI in GIANT at P<5x10^-8^ in the analysis restricted to people of White European heritage, and the per-allele effects on BMI estimated within the same subset of people.

**Exclusion criteria**

The data selection procedure is shown in Figure 2. The initial sample size was 502 528, comprising all UK Biobank participants currently available for analysis. Participants lacking a valid height measurement or whose height was more than 4.56 SD from the mean were excluded. One participant was also excluded for having a seated height >75% of their standing height since this exceeds normal growth (3, 4). Participants without weight information were excluded; however, any participants without a manually recorded weight who had a BMI impedance measure were reintroduced into the dataset. Where both measures of BMI were available, individuals with a difference greater than 4.56 SD between measures were removed.

Individuals lacking full genetic information were also removed, alongside related individuals (the first individual in a related pair, 3^rd^ degree or closer) based on an algorithm applied to the list of all related pairs provided by UK Biobank. This included any individuals who appeared to be highly related to a large number of individuals, derived from the list of individuals excluded from kinship inference. A further set of individuals recommended for exclusion on the grounds of sex-chromosome aneuploidy, sex mismatch or extreme heterozygosity did not exclude any remaining in the analysis at that point. The sample was restricted to those of self-reported White British origin, confirmed genetically as White European using the principal components. Adoptees were excluded, as were any participants missing data on any of the covariates used in the adjustment set, leaving 234 353 UK Biobank participants with valid participant-level data. Of these, longevity data (ie, current age or age at death) were missing for 2495 mothers and 5756 fathers and death preceded the presumed entry to follow-up for a further 221 mothers and 717 fathers. This left an analysis dataset of 231 637 mothers and 227 880 fathers.

**Estimation of the intergenerational associations (IGA) in BMI**

The denominator for an OAI causal estimate should be the mean difference in parental BMI per unit change in offspring BMI. Most studies report mean differences in offspring BMI per unit change in parental BMI, which will not be the same unless the SD of BMI is constant. The two regression coefficients are related by:

β_PBMI~OBMI_ = β_OBMI~PBMI_ * σ^2^_PBMI_ / σ^2^_OBMI_

where β_Y~X_ indicates a mean difference in Y per unit of X, σ^2^_X_ indicates the variance of X and subscripts PBMI and OBMI represent BMI of the parent and offspring, respectively. Cooper et al (5) report SD of BMI of 3.91, 3.05, 5.64 and 4.37 kg/m^2^ for mothers, fathers, female participants and male participants, respectively in the 1958 birth cohort; a cohort born at a similar time and place to UK Biobank and at 45, not dissimilar in age when measured. We therefore used these SD and the equation above to convert their mean differences in offspring BMI per unit of parental BMI to mean differences in parental BMI per unit of offspring BMI for each sex-specific parent offspring combination (all in units of kg/m^2^). This is a slight approximation in the presence of adjustment because the residual error (not reported) should be used in place of the SD. Standard errors of the mean differences were similarly converted by multiplying by the ratio of variances. We took estimates from their models 1 and 4 (their Table 4) to correspond most closely to our unadjusted and adjusted analyses, respectively.

**Estimation of the GRS effect on BMI**

The denominator for a GRS-PGMR estimate should be the mean difference in parental BMI per unit change in the offspring’s GRS. We know that parents and offspring are 50% related, thus, the mean difference in parental BMI per unit change in the offspring’s GRS will be half of the mean difference in offspring BMI per unit change in the offspring’s GRS, with the same SE, if genetic effects on BMI are the same in each generation (6-8). Evidence from GIANT suggests that genetic effects on BMI in men and women are comparable on the scale of sex-specific Z-scores but not on the scale of kg/m^2^. We therefore converted BMI in the UK Biobank participants to sex-specific Z-scores before estimating an effect of the GRS on BMI in men and women together. Between generations, we argue that genetic effects on BMI are likely to be consistent in units of kg/m^2^, since the secular changes in the distribution of BMI are largely environmentally-driven. To calculate the denominators for the GRS-PGMR, we therefore multiplied the single genetic effect of the GRS on the Z-score of BMI by the SD of BMI among UK Biobank participants of the corresponding sex to the parent being analysed, then by 0.5 to allow for the one generation separation. Estimates were repeated with each of the adjustment sets that were used for the numerators.

**Estimation of individual SNP effects on BMI**

According to convention, we refer to PGMR methods involving the combination of single-SNP ratio method IV estimates as summary-level PGMR or, for brevity, “summary-PGMR”. The denominator for each SNP in a summary-PGMR estimate should be the mean difference in parental BMI per BMI-increasing allele of the SNP in the offspring. For the main summary-PGMR estimates, we took the per-allele effect on BMI Z-score reported in GIANT, and its SE. These were multiplied by the separate SD of BMI estimated for UK Biobank men and women to estimate sex-specific effects and their SE in kg/m^2^ per BMI-increasing allele. Finally, the estimates (but not the SE) were multiplied by 0.5 to estimate the increase in parental BMI (kg/m^2^) per BMI-increasing allele in the offspring (6-8). These applied to both sexes of offspring, but the estimate scaled by the SD of BMI in men was used for fathers and the estimate scaled by the SD of BMI in women was used for mothers. Because these denominators were derived from published summary data, it was not possible to estimate them with adjustment.

SNP-specific denominators were also estimated using UK Biobank data for use in PGMR sensitivity analyses we refer to as “summary-PGMR using UK Biobank”. BMI of UK Biobank participants were converted to sex-specific Z-scores and regressed in participants of both sexes against the number of BMI-increasing alleles of the SNP. Standard adjustment sets were applied. These were then converted to sex-specific mean differences in parental BMI (kg/m^2^) per BMI-increasing allele in the offspring in exactly the manner described for SNP effects on BMI in the main summary-PGMR.

**Estimation of OAI and PGMR causal effects of BMI on survival**

OAI and GRS-PGMR estimates were made by the ratio IV method. The numerator was the natural log of the hazard ratio for all-cause mortality per unit of the instrument (ie, offspring BMI or offspring GRS). The denominator was the mean difference in parental BMI (kg/m^2^) per unit of the instrument. This ratio was then exponentiated to produce a hazard ratio for parental mortality per kg/m^2^ of parental BMI. Standard errors and confidence intervals were calculated by the delta method (9, 10) using Taylor expansion (11). The same adjustment was used in corresponding numerators and denominators.

For the summary-PGMR analyses, SNP-specific numerators (log hazard ratios for parental mortality per BMI-increasing allele in the offspring) were estimated as described for the OAI and GRS-PGMR methods, except that the instrument was the SNP-specific genotype. SNP-specific denominators (mean difference in parental BMI (kg/m^2^) per BMI-increasing allele in the offspring) were calculated as described above. The mregger command in Stata was used to combine these SNP-specific estimates as an inverse variance weighted mean (which by convention we refer to as the “IVW estimate”) and to conduct an MR-Egger based -regression. The MR-Egger slope can be interpreted as an MR estimate corrected for pleiotropy, and an MR-Egger intercept that deviates from the null provides evidence for horizontal pleiotropy, if the InSIDE assumption holds (12). The mrmedian and mrmodal commands were used to make summary-PGMR estimates by the inverse variance weighted median and mode methods, which we refer to as “weighted median” and “weighted mode”, respectively.

**Bias component plots**

Bias components for each measured covariate were calculated as the effect of parental BMI on the measured covariate, estimated using offspring BMI or offspring GRS as an instrument (13). Bias components with offspring BMI or offspring GRS as an instrument are proportional to the bias which would result from the omission of the measured covariate from an OAI analysis or a GRS-PGMR analysis, respectively. Estimates were adjusted for offspring date of birth and when the offspring GRS was the instrument, for the first ten principal components. Categorical covariates were dichotomised and analysed with logistic regression (log odds ratios were estimated). Continuous variables were analysed with linear regression. Bias components are only comparable between the two methods, not between sexes or covariates. They were therefore scaled for plotting by the absolute magnitude of the larger of each pair for ease of presentation. Because of the need for comparability between the two methods, all bias components were calculated separately for male and female offspring.

**References**

1. Mitchell R, Hemani G, Dudding T, Paternoster L. UK Biobank Genetic Data: MRC-IEU Quality Control, Version 1. University of Bristol; 2017.

2. Locke AE, Kahali B, Berndt SI, Justice AE, Pers TH, Felix R, et al. Genetic studies of body mass index yield new insights for obesity biology. Nature. 2015;518(7538):197-U401.

3. Wade KH, Carslake D, Sattar N, Davey Smith G, Timpson NJ. BMI and Mortality in UK Biobank: Revised Estimates Using Mendelian Randomization. Obesity. 2018;26(11):1796-806.

4. Tyrrell J, Jones SE, Beaumont R, Astley CM, Lovell R, Yaghootkar H, et al. Height, body mass index, and socioeconomic status: mendelian randomisation study in UK Biobank. BMJ. 2016;352:i582.

5. Cooper R, Hypponen E, Berry D, Power C. Associations between parental and offspring adiposity up to midlife: the contribution of adult lifestyle factors in the 1958 British Birth Cohort Study. Am J Clin Nutr. 2010;92(4):946-53.

6. Joshi PK, Fischer K, Schraut KE, Campbell H, Esko T, Wilson JF. Variants near CHRNA3/5 and APOE have age- and sex-related effects on human lifespan. Nature Communications. 2016;7:11174.

7. Joshi PK, Pirastu N, Kentistou KA, Fischer K, Hofer E, Schraut KE, et al. Genome-wide meta-analysis associates HLA-DQA1/DRB1 and LPA and lifestyle factors with human longevity. Nature Communications. 2017;8(1):910.

8. Wacholder S, Hartge P, Struewing JP, Pee D, McAdams M, Brody L, et al. The kin-cohort study for estimating penetrance. Am J Epidemiol. 1998;148(7):623-30.

9. Hightower AW, Orenstein WA, Martin SM. Recommendations for the use of Taylor series confidence intervals for estimates of vaccine efficacy. Bulletin of the World Health Organization. 1988;66(1):99-105.

10. Pierce BL, Burgess S. Efficient design for Mendelian randomization studies: subsample and 2-sample instrumental variable estimators. American journal of epidemiology. 2013;178(7):1177-84.

11. Thomas DC, Lawlor DA, Thompson JR. Re: Estimation of Bias in Nongenetic Observational Studies Using “Mendelian Triangulation” by Bautista et al. Annals of Epidemiology. 2007;17(7):511-3.

12. Burgess S, Thompson SG. Interpreting findings from Mendelian randomization using the MR-Egger method. European journal of epidemiology. 2017;32(5):377-89.

13. Jackson JW, Swanson SA. Toward a clearer portrayal of confounding bias in instrumental variable applications. Epidemiology (Cambridge, Mass). 2015;26(4):498-504.
