# Supplementary material for: Comparison of intergenerational instrumental variable analyses of body mass index and mortality in UK Biobank
Source: Int J Epidemiol. 2022 Aug 10;52(2):545–61. doi: 10.1093/ije/dyac159 (PMC10114047; doi:10.1093/ije/dyac159)
Supplement: dyac159_Supplementary_Data [file dyac159_supplementary_data.zip › dyac159_Supplementary_Data/ije-2021-09-1429-File007.docx]

**Supplementary Tables for:**

**Comparison of intergenerational instrumental variable analyses of BMI and mortality in UK Biobank**

Ciarrah-Jane Barry, David Carslake, Kaitlin H. Wade, Eleanor Sanderson and George Davey Smith

**Supplementary Table S1.** UK Biobank variables used and their corresponding field ID

| UK Biobank variable | Field ID |
| --- | --- |
| Age when attended assessment centre | 21003 |
| Genetic sex | 22001 |
| Father's age at death | 1807 |
| Mother's age at death | 3526 |
| Body mass index (BMI) | 21001 |
| Year of birth | 34 |
| Month of birth | 52 |
| Qualifications | 6138 |
| Average total household income before tax | 738 |
| Date when attended assessment centre | 53 |
| Alcohol drinker status | 20117 |
| Smoking status | 20116 |
| Current employment status | 6142 |
| Ethnic background | 21000 |
| Standing height | 50 |
| Sitting height | 20015 |
| Weight | 21002 |
| Genetic principal components | 22009 |
| Genetic ethnic grouping | 22006 |
| Body mass index impedance | 23104 |
| Number of days/week of moderate physical activity 10+ minutes | 884 |
| Sex | 31 |
| Mother's age | 1845 |
| Father's age | 2946 |
| Adopted as a child | 1767 |
| Maternal smoking around birth | 1787 |

**Supplementary Table S2.** SNPs used in the PGMR analyses.

|  | Alleles | |  | Most significant analysis | | |  | European sex-combined | | |
| --- | --- | --- | --- | --- | --- | --- | --- | --- | --- | --- |
| SNP | Effect | Other | MSA^a^ | β | SE | P |  | β | SE | P |
| rs1558902 | A | T | ESC | 0.0818 | 0.0031 | 7.51x10⁻¹⁵³ |  | 0.0818 | 0.0031 | 7.51x10⁻¹⁵³ |
| rs6567160 | C | T | ESC | 0.0556 | 0.0036 | 3.93x10⁻⁵³ |  | 0.0556 | 0.0036 | 3.93x10⁻⁵³ |
| rs13021737 | G | A | ESC | 0.0601 | 0.0040 | 1.11x10⁻⁵⁰ |  | 0.0601 | 0.0040 | 1.11x10⁻⁵⁰ |
| rs10938397 | G | A | ESC | 0.0402 | 0.0031 | 3.21x10⁻³⁸ |  | 0.0402 | 0.0031 | 3.21x10⁻³⁸ |
| rs543874 | G | A | ESC | 0.0482 | 0.0039 | 2.62x10⁻³⁵ |  | 0.0482 | 0.0039 | 2.62x10⁻³⁵ |
| rs2207139 | G | A | ESC | 0.0447 | 0.0040 | 4.13x10⁻²⁹ |  | 0.0447 | 0.0040 | 4.13x10⁻²⁹ |
| rs11030104 | A | G | ESC | 0.0414 | 0.0038 | 5.56x10⁻²⁸ |  | 0.0414 | 0.0038 | 5.56x10⁻²⁸ |
| rs3101336 | C | T | ESC | 0.0334 | 0.0031 | 2.66x10⁻²⁶ |  | 0.0334 | 0.0031 | 2.66x10⁻²⁶ |
| rs7138803 | A | G | ESC | 0.0315 | 0.0031 | 8.15x10⁻²⁴ |  | 0.0315 | 0.0031 | 8.15x10⁻²⁴ |
| rs10182181 | G | A | ESC | 0.0307 | 0.0031 | 8.78x10⁻²⁴ |  | 0.0307 | 0.0031 | 8.78x10⁻²⁴ |
| rs3888190 | A | C | ESC | 0.0309 | 0.0031 | 3.14x10⁻²³ |  | 0.0309 | 0.0031 | 3.14x10⁻²³ |
| rs1516725 | C | T | ESC | 0.0451 | 0.0046 | 1.89x10⁻²² |  | 0.0451 | 0.0046 | 1.89x10⁻²² |
| rs12446632 | G | A | ESC | 0.0403 | 0.0046 | 1.48x10⁻¹⁸ |  | 0.0403 | 0.0046 | 1.48x10⁻¹⁸ |
| rs2287019 | C | T | ESC | 0.0360 | 0.0042 | 4.59x10⁻¹⁸ |  | 0.0360 | 0.0042 | 4.59x10⁻¹⁸ |
| rs16951275 | T | C | ESC | 0.0311 | 0.0037 | 1.91x10⁻¹⁷ |  | 0.0311 | 0.0037 | 1.91x10⁻¹⁷ |
| rs3817334 | T | C | ESC | 0.0262 | 0.0031 | 5.15x10⁻¹⁷ |  | 0.0262 | 0.0031 | 5.15x10⁻¹⁷ |
| rs2112347 | T | G | ESC | 0.0261 | 0.0031 | 6.19x10⁻¹⁷ |  | 0.0261 | 0.0031 | 6.19x10⁻¹⁷ |
| rs12566985 | G | A | ESC | 0.0242 | 0.0031 | 3.28x10⁻¹⁵ |  | 0.0242 | 0.0031 | 3.28x10⁻¹⁵ |
| rs3810291 | A | G | ESC | 0.0283 | 0.0036 | 4.81x10⁻¹⁵ |  | 0.0283 | 0.0036 | 4.81x10⁻¹⁵ |
| rs7141420 | T | C | ESC | 0.0235 | 0.0031 | 1.23x10⁻¹⁴ |  | 0.0235 | 0.0031 | 1.23x10⁻¹⁴ |
| rs13078960 | G | T | ESC | 0.0297 | 0.0039 | 1.74x10⁻¹⁴ |  | 0.0297 | 0.0039 | 1.74x10⁻¹⁴ |
| rs10968576 | G | A | ESC | 0.0249 | 0.0033 | 6.61x10⁻¹⁴ |  | 0.0249 | 0.0033 | 6.61x10⁻¹⁴ |
| rs17024393 | C | T | ESC | 0.0658 | 0.0088 | 7.03x10⁻¹⁴ |  | 0.0658 | 0.0088 | 7.03x10⁻¹⁴ |
| rs657452 | A | G | ESC | 0.0227 | 0.0031 | 5.48x10⁻¹³ |  | 0.0227 | 0.0031 | 5.48x10⁻¹³ |
| rs12429545 | A | G | ESC | 0.0334 | 0.0047 | 1.09x10⁻¹² |  | 0.0334 | 0.0047 | 1.09x10⁻¹² |
| rs12286929 | G | A | ESC | 0.0217 | 0.0031 | 1.31x10⁻¹² |  | 0.0217 | 0.0031 | 1.31x10⁻¹² |
| rs13107325 | T | C | ESC | 0.0477 | 0.0068 | 1.83x10⁻¹² |  | 0.0477 | 0.0068 | 1.83x10⁻¹² |
| rs11165643 | T | C | ESC | 0.0218 | 0.0031 | 2.07x10⁻¹² |  | 0.0218 | 0.0031 | 2.07x10⁻¹² |
| rs7903146 | C | T | ESC | 0.0234 | 0.0034 | 1.11x10⁻¹¹ |  | 0.0234 | 0.0034 | 1.11x10⁻¹¹ |
| rs10132280 | C | A | ESC | 0.0230 | 0.0034 | 1.14x10⁻¹¹ |  | 0.0230 | 0.0034 | 1.14x10⁻¹¹ |
| rs17405819 | T | C | ESC | 0.0224 | 0.0033 | 2.07x10⁻¹¹ |  | 0.0224 | 0.0033 | 2.07x10⁻¹¹ |
| rs6091540 | C | T | EW | 0.0297 | 0.0044 | 2.15x10⁻¹¹ |  | 0.0188 | 0.0035 | 8.02x10⁻⁸ |
| rs1016287 | T | C | ESC | 0.0229 | 0.0034 | 2.25x10⁻¹¹ |  | 0.0229 | 0.0034 | 2.25x10⁻¹¹ |
| rs4256980 | G | C | ESC | 0.0209 | 0.0031 | 2.90x10⁻¹¹ |  | 0.0209 | 0.0031 | 2.90x10⁻¹¹ |
| rs17094222 | C | T | ESC | 0.0249 | 0.0038 | 5.94x10⁻¹¹ |  | 0.0249 | 0.0038 | 5.94x10⁻¹¹ |
| rs12401738 | A | G | ESC | 0.0211 | 0.0033 | 1.15x10⁻¹⁰ |  | 0.0211 | 0.0033 | 1.15x10⁻¹⁰ |
| rs7599312 | G | A | ESC | 0.0220 | 0.0034 | 1.17x10⁻¹⁰ |  | 0.0220 | 0.0034 | 1.17x10⁻¹⁰ |
| rs2365389 | C | T | ESC | 0.0200 | 0.0031 | 1.63x10⁻¹⁰ |  | 0.0200 | 0.0031 | 1.63x10⁻¹⁰ |
| rs205262 | G | A | ESC | 0.0221 | 0.0035 | 1.75x10⁻¹⁰ |  | 0.0221 | 0.0035 | 1.75x10⁻¹⁰ |
| rs2820292 | C | A | ESC | 0.0195 | 0.0031 | 1.83x10⁻¹⁰ |  | 0.0195 | 0.0031 | 1.83x10⁻¹⁰ |
| rs12885454 | C | A | ESC | 0.0207 | 0.0033 | 1.94x10⁻¹⁰ |  | 0.0207 | 0.0033 | 1.94x10⁻¹⁰ |
| rs9641123 | C | G | EPB | 0.0286 | 0.0045 | 2.08x10⁻¹⁰ |  | 0.0191 | 0.0038 | 5.00x10⁻⁷ |
| rs12016871^b^ | T | C | ESC | 0.0298 | 0.0047 | 2.29x10⁻¹⁰ |  | 0.0298 | 0.0047 | 2.29x10⁻¹⁰ |
| rs4771122^b^ | G | A | AA | 0.0270 | 0.0043 | 3.41x10⁻¹⁰ |  | 0.0274 | 0.0046 | 2.56x10⁻⁹ |
| rs16851483 | T | G | ESC | 0.0483 | 0.0077 | 3.55x10⁻¹⁰ |  | 0.0483 | 0.0077 | 3.55x10⁻¹⁰ |
| rs1167827 | G | A | ESC | 0.0202 | 0.0033 | 6.33x10⁻¹⁰ |  | 0.0202 | 0.0033 | 6.33x10⁻¹⁰ |
| rs758747 | T | C | ESC | 0.0225 | 0.0037 | 7.47x10⁻¹⁰ |  | 0.0225 | 0.0037 | 7.47x10⁻¹⁰ |
| rs1928295 | T | C | ESC | 0.0188 | 0.0031 | 7.91x10⁻¹⁰ |  | 0.0188 | 0.0031 | 7.91x10⁻¹⁰ |
| rs9925964 | A | G | ESC | 0.0192 | 0.0031 | 8.11x10⁻¹⁰ |  | 0.0192 | 0.0031 | 8.11x10⁻¹⁰ |
| rs11126666 | A | G | ESC | 0.0207 | 0.0034 | 1.33x10⁻⁹ |  | 0.0207 | 0.0034 | 1.33x10⁻⁹ |
| rs2650492 | A | G | ESC | 0.0207 | 0.0035 | 1.92x10⁻⁹ |  | 0.0207 | 0.0035 | 1.92x10⁻⁹ |
| rs6804842 | G | A | ESC | 0.0185 | 0.0031 | 2.48x10⁻⁹ |  | 0.0185 | 0.0031 | 2.48x10⁻⁹ |
| rs12940622 | G | A | ESC | 0.0182 | 0.0031 | 2.49x10⁻⁹ |  | 0.0182 | 0.0031 | 2.49x10⁻⁹ |
| rs7164727 | T | C | AA | 0.0189 | 0.0032 | 3.92x10⁻⁹ |  | 0.0180 | 0.0033 | 6.83x10⁻⁸ |
| rs11847697 | T | C | ESC | 0.0492 | 0.0084 | 3.99x10⁻⁹ |  | 0.0492 | 0.0084 | 3.99x10⁻⁹ |
| rs4740619 | T | C | ESC | 0.0179 | 0.0031 | 4.56x10⁻⁹ |  | 0.0179 | 0.0031 | 4.56x10⁻⁹ |
| rs492400 | C | T | EM | 0.0238 | 0.0041 | 6.78x10⁻⁹ |  | 0.0158 | 0.0031 | 4.17x10⁻⁷ |
| rs13191362 | A | G | ESC | 0.0277 | 0.0048 | 7.34x10⁻⁹ |  | 0.0277 | 0.0048 | 7.34x10⁻⁹ |
| rs3736485 | A | G | ESC | 0.0176 | 0.0031 | 7.41x10⁻⁹ |  | 0.0176 | 0.0031 | 7.41x10⁻⁹ |
| rs17001654 | G | C | ESC | 0.0306 | 0.0053 | 7.76x10⁻⁹ |  | 0.0306 | 0.0053 | 7.76x10⁻⁹ |
| rs11191560 | C | T | ESC | 0.0308 | 0.0053 | 8.45x10⁻⁹ |  | 0.0308 | 0.0053 | 8.45x10⁻⁹ |
| rs2080454 | C | A | AA | 0.0171 | 0.0030 | 8.60x10⁻⁹ |  | 0.0168 | 0.0031 | 6.55x10⁻⁸ |
| rs7715256 | G | T | AA | 0.0168 | 0.0029 | 8.85x10⁻⁹ |  | 0.0163 | 0.0031 | 1.70x10⁻⁷ |
| rs2176040 | A | G | EM | 0.0239 | 0.0042 | 9.99x10⁻⁹ |  | 0.0141 | 0.0031 | 6.06x10⁻⁶ |
| rs1528435 | T | C | ESC | 0.0178 | 0.0031 | 1.20x10⁻⁸ |  | 0.0178 | 0.0031 | 1.20x10⁻⁸ |
| rs2075650 | A | G | ESC | 0.0258 | 0.0045 | 1.25x10⁻⁸ |  | 0.0258 | 0.0045 | 1.25x10⁻⁸ |
| rs1000940 | G | A | ESC | 0.0192 | 0.0034 | 1.28x10⁻⁸ |  | 0.0192 | 0.0034 | 1.28x10⁻⁸ |
| rs2033529 | G | A | ESC | 0.0190 | 0.0033 | 1.39x10⁻⁸ |  | 0.0190 | 0.0033 | 1.39x10⁻⁸ |
| rs11583200 | C | T | ESC | 0.0177 | 0.0031 | 1.48x10⁻⁸ |  | 0.0177 | 0.0031 | 1.48x10⁻⁸ |
| rs7239883 | G | A | EW | 0.0231 | 0.0041 | 1.51x10⁻⁸ |  | 0.0164 | 0.0031 | 1.63x10⁻⁷ |
| rs2836754 | C | T | AA | 0.0169 | 0.0030 | 1.61x10⁻⁸ |  | 0.0164 | 0.0032 | 4.16x10⁻⁷ |
| rs9400239 | C | T | ESC | 0.0188 | 0.0033 | 1.61x10⁻⁸ |  | 0.0188 | 0.0033 | 1.61x10⁻⁸ |
| rs10733682 | A | G | ESC | 0.0174 | 0.0031 | 1.83x10⁻⁸ |  | 0.0174 | 0.0031 | 1.83x10⁻⁸ |
| rs11688816 | G | A | ESC | 0.0172 | 0.0031 | 1.89x10⁻⁸ |  | 0.0172 | 0.0031 | 1.89x10⁻⁸ |
| rs11057405 | G | A | ESC | 0.0307 | 0.0055 | 2.02x10⁻⁸ |  | 0.0307 | 0.0055 | 2.02x10⁻⁸ |
| rs9914578 | G | C | AA | 0.0201 | 0.0036 | 2.07x10⁻⁸ |  | 0.0201 | 0.0038 | 8.99x10⁻⁸ |
| rs977747 | T | G | AA | 0.0168 | 0.0030 | 2.18x10⁻⁸ |  | 0.0167 | 0.0031 | 8.65x10⁻⁸ |
| rs2121279 | T | C | ESC | 0.0245 | 0.0044 | 2.31x10⁻⁸ |  | 0.0245 | 0.0044 | 2.31x10⁻⁸ |
| rs29941 | G | A | ESC | 0.0182 | 0.0033 | 2.41x10⁻⁸ |  | 0.0182 | 0.0033 | 2.41x10⁻⁸ |
| rs11727676 | T | C | ESC | 0.0358 | 0.0064 | 2.55x10⁻⁸ |  | 0.0358 | 0.0064 | 2.55x10⁻⁸ |
| rs3849570 | A | C | ESC | 0.0188 | 0.0034 | 2.60x10⁻⁸ |  | 0.0188 | 0.0034 | 2.60x10⁻⁸ |
| rs6477694 | C | T | ESC | 0.0174 | 0.0031 | 2.67x10⁻⁸ |  | 0.0174 | 0.0031 | 2.67x10⁻⁸ |
| rs9374842 | T | C | EPB | 0.0229 | 0.0041 | 2.67x10⁻⁸ |  | 0.0187 | 0.0035 | 9.67x10⁻⁸ |
| rs4787491 | G | A | EPB | 0.0217 | 0.0039 | 2.70x10⁻⁸ |  | 0.0159 | 0.0034 | 2.24x10⁻⁶ |
| rs1441264 | A | G | AA | 0.0172 | 0.0031 | 2.96x10⁻⁸ |  | 0.0175 | 0.0032 | 6.04x10⁻⁸ |
| rs7899106 | G | A | ESC | 0.0395 | 0.0071 | 2.96x10⁻⁸ |  | 0.0395 | 0.0071 | 2.96x10⁻⁸ |
| rs2176598 | T | C | ESC | 0.0198 | 0.0036 | 2.97x10⁻⁸ |  | 0.0198 | 0.0036 | 2.97x10⁻⁸ |
| rs2245368 | C | T | ESC | 0.0317 | 0.0057 | 3.19x10⁻⁸ |  | 0.0317 | 0.0057 | 3.19x10⁻⁸ |
| rs17203016 | G | A | AA | 0.0211 | 0.0038 | 3.41x10⁻⁸ |  | 0.0210 | 0.0039 | 8.15x10⁻⁸ |
| rs17724992 | A | G | ESC | 0.0194 | 0.0035 | 3.42x10⁻⁸ |  | 0.0194 | 0.0035 | 3.42x10⁻⁸ |
| rs7243357 | T | G | ESC | 0.0217 | 0.0040 | 3.86x10⁻⁸ |  | 0.0217 | 0.0040 | 3.86x10⁻⁸ |
| rs16907751 | C | T | EM | 0.0474 | 0.0086 | 3.89x10⁻⁸ |  | 0.0350 | 0.0066 | 1.26x10⁻⁷ |
| rs1808579 | C | T | ESC | 0.0167 | 0.0031 | 4.17x10⁻⁸ |  | 0.0167 | 0.0031 | 4.17x10⁻⁸ |
| rs13201877 | G | A | AA | 0.0236 | 0.0043 | 4.29x10⁻⁸ |  | 0.0233 | 0.0045 | 2.35x10⁻⁷ |
| rs2033732 | C | T | ESC | 0.0192 | 0.0035 | 4.89x10⁻⁸ |  | 0.0192 | 0.0035 | 4.89x10⁻⁸ |
| rs9540493 | A | G | EPB | 0.0210 | 0.0038 | 4.97x10⁻⁸ |  | 0.0172 | 0.0033 | 1.42x10⁻⁷ |
| rs1460676 | C | T | AA | 0.0209 | 0.0038 | 4.98x10⁻⁸ |  | 0.0197 | 0.0040 | 8.98x10⁻⁷ |
| rs6465468 | T | G | EW | 0.0245 | 0.0045 | 4.98x10⁻⁸ |  | 0.0166 | 0.0035 | 2.32x10⁻⁶ |

*SNP = single nucleotide polymorphism, BMI = body mass index, MSA = most significant analysis, β= effect size in standard deviations of BMI per effect allele, SE = standard error. Data are taken from the GIANT consortium. (1) SNPs were included in the analysis at P<5x10^-8^; the criterion was met by 97 SNPs in the most significant analysis and 77 in the European sex-combined analysis.*

*^a^European sex-combined (ESC), European men (EM), European women (EW), European population-based (EPB) or all ancestries (AA).*

*^b^Data on rs12016871 were not available in UK Biobank so rs4771122 (which is in close linkage disequilibrium with rs12016871) was used instead.*

**Supplementary Table S3.** Mean differences in parental BMI (kg/m^2^) per kg/m^2^ of offspring BMI from the literature.

| Setting | Age at BMI measurement | Mean (SD) of BMI, kg/m^2^ | Adjustment | MD (SE) in parental BMI (kg/m^2^) per kg/m^2^ of offspring BMI |
| --- | --- | --- | --- | --- |
| 1958 National Child Development Study; 4695 girls and 4651 boys born in the UK in 1958. (2) | P: In 1969 o: 44-45 years | M: 24.04 (3.91) F: 24.70 (3.05) d: 27.00 (5.64) s: 27.84 (4.37) | Parental age | Md: 0.168 (0.012) Ms: 0.192 (0.014) Fd: 0.079 (0.009) Fs: 0.136 (0.011) |
|  |  |  | Parental age, diet, tv, activity, smoking, alcohol. Father's employment, education. Offspring's employment, education. | Md: 0.139 (0.011) Ms: 0.168 (0.014) Fd: 0.064 (0.008) Fs: 0.132 (0.010) |
| Swedish conscripts; 68,886 father-son pairs. Average DOB for sons 1977.8. (3) | F: 16-25 years s: 16-25 years | F: 21.2 s: 22.4 | Father's age, conscription office. Offspring's age, conscription office, DOB. | Fs: 0.214 (0.003) |
|  |  |  | Father's age, conscription office, education, employment. Offspring's age, conscription office, DOB. | Fs: 0.210 (0.003) |
| HUNT study; 32,452 mother-offspring and 27,747 father-offspring pairs measured 1984-2008 in Nord-Trøndelag county, Norway. (4) | M: 47.4 years F: 48.54 years o: 28.48 years (means) | M: 25.26 F: 25.48 o: 24.08 | Parent's age, sex, HUNT survey, DOB, alcohol, education, employment, exercise, smoking. Offspring's age, sex, HUNT survey, smoking. Parent's partner's employment. | Md: 0.240 (0.005) Ms: 0.307 (0.007) Fd: 0.172 (0.004) Fs: 0.220 (0.005) |
| Cross-sectional household survey in Tumbes, Peru in 2012-2017. N=168 (Md), 230 (Ms), 142 (Fd) & 195 (Fs) pairs. (5) | M: 54 years F: 59 years o: 29 years (means) | M: 29.1 (4.8) F: 26.6 (4.4) d: 25.6 (4.7) s: 25.1 (4.1) | None | Md: 0.104 (0.061) Ms: 0.274 (0.070) Fd: 0.167 (0.056) Fs: 0.230 (0.071) |
|  |  |  | Household's wealth, village. Offspring's age, education, activity. | Md: 0.115 (0.059) Ms: 0.274 (0.073) Fd: 0.219 (0.054) Fs: 0.299 (0.071) |
| 263 girls and 248 boys recruited in 2001 in London & SE England. (6) | P: In 2001 d: 5-21 years s: 5-21 years | M of d: 25.3 (5.2) M of s: 25.5 (4.7) F of d: 26.3 (4.1) F of s: 26.5 (3.7) d: 19.8 (3.8) s: 18.9 (3.9) | None | Md: 0.534 (0.093) Ms: 0.459 (0.085) Fd: 0.142 (0.082) Fs: 0.200 (0.070) |
| Mater university study; 3340 children born 1981-1984 in Australia. (7) | P: At offspring birth o: 14 years | M: 22.0 (4.1) F: 23.7 (4.7) d: 21.0 (4.0) s: 20.3 (3.6) | Offspring sex. Other parent's BMI. | Mo: 0.392 (0.022) Fo: 0.298 (0.027) |
|  |  |  | Parent's income, education, smoking. Offspring sex. Other parent's BMI. Mother's age at offspring birth, parity. | Mo: 0.391 (0.022) Fo: 0.296 (0.027) |
| School children in Copenhagen, Denmark born 1957-1960. N=2377 (Md), 2800 (Ms), 1386 (Fd) & 1617 (Fs) pairs. (8) | P: 7 years o: 7 years | M: 15.5 (1.3) F: 15.4 (1.1) d: 15.4 (1.4) s: 15.5 (1.25) | Parent's age at offspring birth. Other parent's BMI. | Md: 0.306 (0.019) Ms: 0.370 (0.018) Fd: 0.196 (0.020) Fs: 0.211 (0.023) |
| ALSPAC; children born in Avon, England in 1991-1992. 6815 mothers and 4955 fathers. (9) | P: At offspring birth o: 7.5 years | M: 22.9 (3.7) F: 25.1 (3.2) o: 16.2 (2.0) | Parent's age. Offspring sex, age. | Mo: 0.534 (0.025) Fo: 0.397 (0.023) |
| PROBIT; 11,353 to 12,181 Belarusian children born 1996-1997. (10) | M: 31.7 years F: 34.1 years o: 6.5 years | M:24.5 (4.4) F: 25.7 (3.3) d: 15.4 (1.7) s: 15.7 (1.6) | Parent's age at offspring birth. Offspring's age, sex. Randomisation arm. | Mo: 0.533 (0.041) Fo: 0.380 (0.026) |
|  |  |  | Parent's age at offspring birth, social class, education, smoking. Offspring's age, sex, rural/urban, family size. Randomisation arm. Other parent smoking, BMI. | Mo: 0.560 (0.048) Fo: 0.400 (0.026) |
| Lifeways; 454 mothers and 191 fathers of children born in Ireland in 2002-2003. (11) | P: At offspring birth o: 5 years | M: 23.7 (3.8) F: 26.3 (3.8) d: 16.6 (1.8) s: 16.6 (1.7) | None | Mo: 0.432 (0.124) Fo: 0.238 (0.155) |
| MoBa; 29,216 children born in Norway in 1999-2009. (12) | M: 33.3 years F: 35.9 years (means) o: 3 years | M: 24.1 (4.2) F: 25.8 (3.3) o: 16.1 (1.5) | None | Mo: 0.265 (0.015) Fo: 0.208 (0.013) |
|  |  |  | Parent's education, smoking. Mother's smoking. Offspring's siblings, day care, breastfed, activity, diet. Other parent's BMI. | Mo: 0.220 (0.015) Fo: 0.176 (0.013) |

*BMI = body mass index, SD = standard deviation, MD = mean difference, SE = standard error, P = parents, M = mothers, F = fathers, o = offspring, d = daughters, s = sons.*

*Where multiple cohorts and/or adjustment sets were available, we chose those closest to the UK Biobank data and our adjustment variables. MD were often reported with the exposure or outcome in SD units and/or with offspring BI as the outcome. These were converted using reported SD to make parental BMI the outcome and kg/m^2^ the units of BMI in exposure and outcome.*

**Supplementary Table S4.** Hazard ratios for all-cause mortality per kg/m^2^ of BMI using PGMR sensitivity analyses.

|  |  | Hazard ratio (95% CI) using method: | | | | |
| --- | --- | --- | --- | --- | --- | --- |
| Parent | Adjustment | IVW estimate | MR-Egger (intercept) | MR-Egger (estimate) | Weighted median | Weighted mode |
| *Summary-level proxy-genotype Mendelian randomization with independent samples (summary-PGMR):* | | | | | | |
| Mothers | Unadjusted^a^ | 1.03 (1.01, 1.04) | 1.00 (1.00, 1.01) | 1.00 (0.95, 1.06) | 1.02 (1.00, 1.04) | 1.01 (0.98, 1.04) |
| Fathers | Unadjusted^a^ | 1.05 (1.03, 1.06) | 1.00 (1.00, 1.01) | 1.02 (0.96, 1.08) | 1.04 (1.01, 1.06) | 1.03 (1.00, 1.07) |
| Mothers | Adjusted^b^ | 1.02 (1.01, 1.04) | 1.00 (1.00, 1.00) | 1.01 (0.95, 1.06) | 1.02 (1.00, 1.04) | 1.01 (0.98, 1.04) |
| Fathers | Adjusted^b^ | 1.04 (1.02, 1.05) | 1.00 (1.00, 1.00) | 1.02 (0.96, 1.08) | 1.04 (1.01, 1.06) | 1.03 (1.00, 1.07) |
| *Summary-level proxy-genotype Mendelian randomization without independent samples (summary-PGMR using UK Biobank):* | | | | | | |
| Mothers | Unadjusted^a^ | 1.03 (1.02, 1.05) | 1.00 (1.00, 1.00) | 1.02 (0.97, 1.07) | 1.03 (1.00, 1.05) | 1.01 (0.98, 1.04) |
| Fathers | Unadjusted^a^ | 1.06 (1.05, 1.08) | 1.00 (1.00, 1.00) | 1.06 (1.01, 1.12) | 1.06 (1.03, 1.08) | 1.05 (1.02, 1.09) |
| Mothers | Adjusted^b^ | 1.03 (1.01, 1.04) | 1.00 (1.00, 1.00) | 1.02 (0.97, 1.07) | 1.02 (0.99, 1.04) | 1.01 (0.98, 1.05) |
| Fathers | Adjusted^b^ | 1.06 (1.04, 1.07) | 1.00 (1.00, 1.00) | 1.06 (1.01, 1.12) | 1.05 (1.02, 1.08) | 1.05 (1.01, 1.08) |

*BMI = body mass index; CI = confidence interval; SNP = single nucleotide polymorphism. Analyses used the 97 SNPs identified as genome-wide significant at P<5x10^-8^ in the “most significant analysis” of the GIANT consortium. (1) The summary-PGMR analyses also used BMI-SNP associations from GIANT. N = 231 637 mothers and 227 880 fathers of 233 361 UK Biobank participants.*

*^a^Unadjusted analyses were adjusted for the first ten genetic principal components and for secular trends (offspring date of birth).*

^b^*Adjusted analyses were additionally adjusted for highest level of education attained, current employment status, smoking status, alcohol intake, physical activity, age when attended assessment centre and average household income before tax.*

**Supplementary Table S5.** Sensitivity analysis using only the 77 SNPs genome-wide significant at P<5x10^-8^ in men and women of white European ancestry.

|  |  |  | Unadjusted model^a^ | | |  | Adjusted model^b^ | | |
| --- | --- | --- | --- | --- | --- | --- | --- | --- | --- |
| Parent | Offspring | N | HR (95% CI) | P-value | SE(Ln(HR)) |  | HR (95% CI) | P-value | SE(Ln(HR)) |
| *Proxy-genotype Mendelian randomization using genetic risk scores (GRS-PGMR):* | | | | | | | | |  |
| Mothers | Both | 231 637 | 1.03 (1.01, 1.05) | <0.001 | 0.008 |  | 1.02 (1.01, 1.04) | 0.005 | 0.009 |
| Fathers | Both | 227 880 | 1.06 (1.04, 1.08) | <0.001 | 0.009 |  | 1.05 (1.03, 1.07) | <0.001 | 0.009 |
| *IVW estimates from summary-level proxy-genotype Mendelian randomization with independent samples (summary-PGMR):* | | | | | | | | |  |
| Mothers | Both | 231 637 | 1.02 (1.01, 1.04) | <0.001 | 0.007 |  | 1.02 (1.01, 1.04) | 0.004 | 0.007 |
| Fathers | Both | 227 880 | 1.05 (1.03, 1.07) | <0.001 | 0.008 |  | 1.04 (1.03, 1.06) | <0.001 | 0.008 |

*SNP = single nucleotide polymorphism; HR = hazard ratio; CI = confidence interval**; SE = standard error; BMI = body mass* index. Hazard ratios are for all-cause mortality per kg/m^2^ of BMI using offspring genotype as an instrument for parent’s BMI. Offspring are UK Biobank pa*rticipants.*

*^a^Unadjusted PGMR analyses were adjusted for the first ten genetic principal components and for secular trends (offspring date of birth).*

^b^*Adjusted analyses were additionally adjusted for highest level of education attained, current employment status, smoking status, alcohol intake, physical activity, age when attended assessment centre and average household income before tax.*

**Supplementary Table S6.** Sensitivity analyses with alternative imputation of entry to follow-up.

|  |  | Imputation of entry to follow-up for deceased parents from parental age of surviving parents | | | | | | | |
| --- | --- | --- | --- | --- | --- | --- | --- | --- | --- |
|  |  | Mean (main analysis) | |  | 5^th^ percentile | |  | 95^th^ percentile | |
| Parent | Offspring | N | HR (95% CI) |  | N | HR (95% CI) |  | N | HR (95% CI) |
| *Offspring BMI as instrument (OAI):* | | | | | | | | | |
| Mothers | Daughters | 120 489 | 1.11 (1.09, 1.14) |  | 120 584 | 1.11 (1.09, 1.14) |  | 119 867 | 1.11 (1.09, 1.14) |
| Mothers | Sons | 111 148 | 1.08 (1.06, 1.10) |  | 111 257 | 1.08 (1.06, 1.10) |  | 110 595 | 1.08 (1.06, 1.10) |
| Mothers | Both^a^ | 231 637 | 1.10 (1.08, 1.11) |  | 231 841 | 1.10 (1.08, 1.11) |  | 230 462 | 1.10 (1.08, 1.11) |
| Fathers | Daughters | 118 181 | 1.23 (1.16, 1.29) |  | 118 437 | 1.23 (1.16, 1.29) |  | 117 150 | 1.22 (1.16, 1.29) |
| Fathers | Sons | 109 699 | 1.12 (1.10, 1.14) |  | 109 998 | 1.12 (1.10, 1.14) |  | 108 802 | 1.12 (1.10, 1.15) |
| Fathers | Both^a^ | 227 880 | 1.13 (1.11, 1.16) |  | 228 435 | 1.13 (1.11, 1.16) |  | 225 952 | 1.14 (1.11, 1.16) |
| *Proxy-genotype Mendelian randomization using genetic risk scores (GRS-PGMR):* | | | | | | | | | |
| Mothers | Both | 231 637 | 1.03 (1.01, 1.04) |  | 231 841 | 1.03 (1.01, 1.04) |  | 230 462 | 1.03 (1.01, 1.04) |
| Fathers | Both | 227 880 | 1.05 (1.03, 1.07) |  | 228 435 | 1.05 (1.03, 1.07) |  | 225 952 | 1.05 (1.03, 1.07) |
| *IVW estimates from summary-level proxy-genotype Mendelian randomization with independent samples (summary-PGMR):* | | | | | | | | | |
| Mothers | Both | 231 637 | 1.02 (1.01, 1.04) |  | 231 841 | 1.02 (1.01, 1.03) |  | 230 462 | 1.02 (1.01, 1.04) |
| Fathers | Both | 227 880 | 1.04 (1.02, 1.05) |  | 228 435 | 1.04 (1.02, 1.05) |  | 225 952 | 1.04 (1.03, 1.05) |

*HR = hazard ratio; CI = confidence interval; BMI = body mass index. Hazard ratios are for all-cause mortality per kg/m^2^ of BMI using offspring BMI or offspring genotype as an instrument for parent’s BMI. Analyses were adjusted for secular trends (offspring date of birth), highest level of education attained, current employment status, smoking status, alcohol intake, physical activity, age when attended assessment centre and average household income before tax. PGMR analyses were additionally adjusted for the first ten genetic principal components. Offspring are UK Biobank participants.*

*^a^Estimates from sons and daughters were combined in meta-analyses, which indicated substantial heterogeneity between them.*

**Supplementary Table S7.** Proportionality of hazards and age-split hazard ratios.

|  |  |  | HR (95% CI) | | |
| --- | --- | --- | --- | --- | --- |
| Parent | Offspring | P_PH_^a^ | All follow-up^b^ | <70years old^c^ | >70years old^c^ |
| *Offspring BMI as instrument (OAI):* | | | |  |  |
| Mothers | Daughters | 0.011 | 1.11 (1.09, 1.14) | 1.15 (1.12, 1.18) | 1.10 (1.07, 1.12) |
| Mothers | Sons | 0.007 | 1.08 (1.06, 1.10) | 1.13 (1.10, 1.16) | 1.06 (1.04, 1.08) |
| Fathers | Daughters | 0.002 | 1.23 (1.16, 1.29) | 1.29 (1.21, 1.39) | 1.17 (1.11, 1.23) |
| Fathers | Sons | <0.001 | 1.12 (1.10, 1.14) | 1.16 (1.13, 1.19) | 1.09 (1.07, 1.11) |
| *Proxy-genotype Mendelian randomization using genetic risk scores (GRS-PGMR):* | | | | | |
| Mothers | Both | 0.146 | 1.03 (1.01, 1.04) | 1.06 (1.03, 1.09) | 1.01 (0.99, 1.03) |
| Fathers | Both | 0.006 | 1.05 (1.03, 1.07) | 1.08 (1.05, 1.11) | 1.03 (1.00, 1.05) |

*BMI = body mass index (kg/m^2^); HR = hazard ratio; CI = confidence interval Hazard ratios are for all-cause mortality per kg/m^2^ of BMI using offspring BMI or offspring genotype as an instrument for parent’s BMI. Analyses were adjusted for secular trends (offspring date of birth), highest level of education attained, current employment status, smoking status, alcohol intake, physical activity, age when attended assessment centre and average household income before tax. PGMR analyses were additionally adjusted for the first ten genetic principal components. Offspring are UK Biobank participants.*

*^a^P-value for correlation between the Schoenfeld residuals for BMI and Ln(age).*

*^b^From the analysis including all follow-up (Table 6)*

*^c^From an analysis in which follow-up was split into time before and after the parent’s 70^th^ birthday. The same denominator (instrument-exposure association) was assumed to apply in each age period.*

**References**

1. Locke AE, Kahali B, Berndt SI, Justice AE, Pers TH, Felix R, et al. Genetic studies of body mass index yield new insights for obesity biology. Nature. 2015;518(7538):197-U401.

2. Cooper R, Hyppönen E, Berry D, Power C. Associations between parental and offspring adiposity up to midlife: the contribution of adult lifestyle factors in the 1958 British Birth Cohort Study. Am J Clin Nutr. 2010;92(4):946-53.

3. Wade KH, Carslake D, Tynelius P, Davey Smith G, Martin RM. Variation of all-cause and cause-specific mortality with body mass index in one million Swedish parent-son pairs: An instrumental variable analysis. PLoS Med. 2019;16(8).

4. Carslake D, Davey Smith G, Gunnell D, Davies N, Nilsen T, Romundstad P. Confounding by ill health in the observed association between BMI and mortality: evidence from the HUNT Study using offspring BMI as an instrument. Int J Epidemiol. 2017;47(3):760-70.

5. Carrillo-Larco RM, Bernabe-Ortiz A, Rosas V, Sacksteder KA, Diez-Canseco F, Cardenas MK, et al. Parental body mass index and blood pressure are associated with higher body mass index and blood pressure in their adult offspring: a cross-sectional study in a resource-limited setting in northern Peru. Trop Med Int Health. 2018;23(5):533-40.

6. Devakumar D, Grijalva-Eternod C, Cortina-Borja M, Williams J, Fewtrell M, Wells J. Disentangling the associations between parental BMI and offspring body composition using the four-component model. Am J Hum Biol. 2016;28(4):524-33.

7. Lawlor DA, Davey Smith G, O'Callaghan M, Alati R, Mamun AA, Williams GM, et al. Epidemiologic evidence for the fetal overnutrition hypothesis: Findings from the Mater-University Study of Pregnancy and its outcomes. Am J Epidemiol. 2007;165(4):418-24.

8. Ajslev TA, Ängquist L, Silventoinen K, Baker JL, Sørensen TIA. Trends in parent-child correlations of childhood body mass index during the development of the obesity epidemic. PLoS ONE. 2014;9(10).

9. Davey Smith G, Steer C, Leary S, Ness A. Is there an intrauterine influence on obesity? Evidence from parent child associations in the Avon Longitudinal Study of Parents and Children (ALSPAC). Arch Dis Child. 2007;92(10):876-80.

10. Patel R, Martin RM, Kramer MS, Oken E, Bogdanovich N, Matush L, et al. Familial associations of adiposity: Findings from a cross-sectional study of 12,181 parental-offspring trios from Belarus. PLoS ONE. 2011;6(1).

11. Murrin CM, Kelly GE, Tremblay RE, Kelleher CC. Body mass index and height over three generations: evidence from the Lifeways cross-generational cohort study. BMC Public Health. 2012;12.

12. Fleten C, Nystad W, Stigum H, Skjærven R, Lawlor DA, Davey Smith G, et al. Parent-offspring body mass index associations in the Norwegian Mother and Child Cohort Study: A family-based approach to studying the role of the intrauterine environment in childhood adiposity. Am J Epidemiol. 2012;176(2):83-92.
